# SHP2 inhibitors maintain TGFβ signalling through SMURF2 inhibition

**DOI:** 10.1038/s41698-023-00486-6

**Published:** 2023-12-15

**Authors:** Xianning Lai, Sarah Kit Leng Lui, Hiu Yan Lam, Yuta Adachi, Wen Jing Sim, Natali Vasilevski, Nicola J. Armstrong, Stephanie Claire Bridgeman, Nathan Michael Main, Tuan Zea Tan, Janina E. E. Tirnitz-Parker, Jean Paul Thiery, Hiromichi Ebi, Alan Prem Kumar, Pieter Johan Adam Eichhorn

**Affiliations:** 1 Cancer Science Institute of Singapore, National University of Singapore, Singapore, 117599 Singapore; 2 NUS Center for Cancer Research (N2CR), Yong Loo Lin School of Medicine, National University of Singapore, 117597 Singapore, Singapore; 3 Department of Pharmacology, Yong Loo Lin School of Medicine, National University of Singapore, Singapore, 117600 Singapore; 4 Division of Molecular Therapeutics, Aichi Cancer Center Research Institute, Nagoya, Aichi 464-8681 Japan; 5 Division of Advanced Cancer Therapeutics, Nagoya University Graduate School of Medicine, Nagoya, Aichi 466-8650 Japan; 6 Institute of Molecular and Cell Biology, A*STAR, Singapore, 138672 Singapore; 7 Curtin Medical School, Faculty of Health Sciences, Curtin University, Bentley, WA 6102 Australia; 8 Curtin Health Innovation Research Institute and Faculty of Health Sciences, Curtin University, Bentley, WA 6102 Australia; 9 School of Electrical Engineering, Computing and Mathematical Sciences, Faculty of Science and Engineering, Curtin University, Bentley, WA 6102 Australia; 10 Guangzhou Laboratory, Guangzhou International Bio Island, Haizhu District, Guangzhou, Guangdong, 510530 China

**Keywords:** Phosphorylation, Cancer therapeutic resistance

## Abstract

Despite the promising antitumor activity of SHP2 inhibitors in RAS-dependent tumours, overall responses have been limited by their narrow therapeutic window. Like with all MAPK pathway inhibitors, this is likely the result of compensatory pathway activation mechanisms. However, the underlying mechanisms of resistance to SHP2 inhibition remain unknown. The E3 ligase SMURF2 limits TGFβ activity by ubiquitinating and targeting the TGFβ receptor for proteosome degradation. Using a functional RNAi screen targeting all known phosphatases, we identify that the tyrosine phosphatase SHP2 is a critical regulator of TGFβ activity. Specifically, SHP2 dephosphorylates two key residues on SMURF2, resulting in activation of the enzyme. Conversely, SHP2 depletion maintains SMURF2 in an inactive state, resulting in the maintenance of TGFβ activity. Furthermore, we demonstrate that depleting SHP2 has significant implications on TGFβ-mediated migration, senescence, and cell survival. These effects can be overcome through the use of TGFβ-targeted therapies. Consequently, our findings provide a rationale for combining SHP2 and TGFβ inhibitors to enhance tumour responses leading to improved patient outcomes.

## Introduction

Clinical observations indicate that a considerable proportion of patients display robust responses to targeted therapies. However, response rates to these agents are variable and lessons learned from these targeted therapy paradigms inform us that sensitive cancers will eventually become resistant to these agents. Primary resistance is permutated by co-existing genetic alterations in malignant cells that provide these cells a clonal advantage to escape therapeutic pressure. Cancer cells can also acquire genomic alterations over time through a variety of mutational processes that limit therapeutic responses^1^. Along with genetic determinants of therapy resistance, tumourigenic cells may achieve non-mutational forms of resistance or drug tolerance through phenotype switching or changes in cellular plasticity^2–4^. Another characteristic of drug-tolerant tumour populations is cell cycle restriction defined by quiescence or senescence^5^. Senescence induction can occur via multiple mechanisms, including DNA damage, excessive oncogenic signalling, telomere shortening or exposure to a variety of stress signals^6^. Likewise, radiotherapy and various chemotherapies can induce cancer senescence. Importantly, these residual senescent cells constitute a reservoir of tumourigenic cells that may lead to therapy evasion when they acquire the ability to re-enter the cell cycle either through the gain of secondary genetic mutations, epigenetic alterations, or changes in the microenvironment.

The Src homology 2 (SH2) domain-containing protein tyrosine phosphatase-2 (SHP2, encoded by *PTPN11*) is a non-receptor tyrosine phosphatase that functions downstream of multiple receptor tyrosine kinases (RTKs) to promote the activation of the RAS/RAF/ERK/MAPK pathway. The characteristic SH2 domains are critical for SHP2 engagement of phosphotyrosine residues on RTKs and various signalling molecules. More recently, it has been demonstrated that SHP2 is essential for the activation of RAS and indispensable for the establishment of KRAS mutant non-small cell lung cancer^7,8^. Mechanistically, RAS can be phosphorylated at tyrosine 32 by the tyrosine kinase c-SRC within the switch I region. This event results in increased GAP binding and GTP hydrolysis, limiting downstream RAF activation. Conversely, SHP2 dephosphorylates tyrosine 32, resulting in activation of downstream RAS/RAF/ERK/MAPK signalling. In normal cells, SHP2 would function to reactivate RAS through dephosphorylation to initiate a new RAS GTPase cycle. Considering its role in RAS activation, it is unsurprising that SHP2 is frequently mutated in several diseases including cancer. Autosomal dominant activating mutations in PTPN11 are linked with driving human RASopathies including Noonan Syndrome^9^. Similarly, somatic gain of function SHP2 mutations drive several haematological malignancies. Additionally, altered SHP2 activity has been identified as a mechanism of resistance to several tyrosine kinase inhibitors through reactivation of MAPK signalling. As a result of the oncogenic activity of SHP2, several SHP2 inhibitors have been developed and evaluated in clinical trials for the treatment of solid tumours including KRAS mutant non-small cell lung cancer. Nevertheless, long-term tumour responses were negligible.

Transforming growth factor β (TGFβ) forms part of a superfamily of evolutionary conserved cytokines that play key roles in both embryonic development and subsequently in maintaining tissue homoeostasis in adult tissues^10–13^. However, TGFβ plays a dichotomous role in cancer biology, acting as an oncogenic driver in later stage tumours by maintaining cell survival, epithelial-mesenchymal transition (EMT), and immune surveillance^14–17^. Conversely, TGFβ functions as an early tumour suppressor by inducing a cytostatic response mediated by cyclin-dependent kinase inhibitors p15^INK4b^, p21, and p27. Similarly, TGFβ can regulate the activity of oncogenes c-MYC, TERT, and induce ROS production. Although context-specific, the prolonged alteration in any of these factors can result in TGFβ -mediated induced senescence.

TGFβ ligand binding induces the formation of a tetrameric complex consisting of two TGFβ receptor I (TβRI) subunits and two TGFβ receptor II (TβRII) subunits^18^. This tetrameric complex enhances intercellular signalling by phosphorylating the Receptor-regulated SMADs (R-SMADs), specifically SMAD2 and SMAD3^10,12^. The now activated R-SMADs associate with a common-partner, the co-SMAD, SMAD4. The SMAD2/4 or SMAD3/4 complex can then enter the nucleus and bind to the conserved SMAD binding element (SBE) sequences driving transcription of a variety of genes^11,12,19^. To maintain an equilibrium and to ensure that extracellular signalling generates the desired intracellular responses, a number of transcriptionally-mediated negative feedback loops exist to limit hyperactivation of the pathway.

For the TGFβ pathway, SMAD complexes induce the transcription of both TGFβ target genes SMAD7 and USP26^20–23^. The deubiquitinating enzyme USP26 deubiquitinates and stabilizes SMAD7 allowing SMAD7 to act as scaffold to recruit the HECT-E3 ligase SMURF2 to the TGFβ receptor complex, facilitating ubiquitin-mediated proteasomal degradation of the receptor complex and attenuating TGFβ signalling^20–23^. Independent of acting as a scaffold, SMAD7 also induces the ubiquitin ligase activity of SMURF2. The SMURF2 protein comprises a C2 domain, three WW domains and a C-terminal HECT domain^24^. To maintain SMURF2 in an inhibitory state and limit unnecessary activity towards its substrates, the N-terminal C2 domain interacts with the C-terminal HECT domain inhibiting ubiquitin thioester bond formation of its catalytic cysteine residue^25^. SMAD7 binding to the WW3 domain of SMURF2 unfastens C2 from the HECT domain, releasing the inhibitory interactions between these two domains and eventually freeing up the catalytic cysteine for subsequent ligase activity towards its substrates^25,26^. Recently, we have demonstrated that the tyrosine kinase c-SRC successively plays a role in this process by phosphorylating SMURF2 at Tyr314 in the WW3 domain and Tyr434 in the HECT domain. Phosphorylation at Tyr314 prevents SMAD7 binding as the negative phosphorylation charge on Tyr314 attracts adjacent arginine residues Arg306 and Arg321, forming a salt-bridge interaction changing the physical nature of the domain. Supplemental to this, phosphorylation of Tyr434 enhances the intramolecular interactions between the C2 domain and the HECT domain^26^. The combined effect of c-SRC-mediated phosphorylation at Tyr314 and Tyr434 is to maintain SMURF2 in a closed inactive conformation^26^. However, the mechanism of reactivation of SMURF2 through dephosphorylation remains unknown. Here, we demonstrate that SHP2 dephosphorylates SMURF2 at Tyr314 and Tyr434 to activate SMURF2 and downregulate TGFβ signalling. Furthermore, we demonstrate that genetic or chemical inhibition of SHP2 activates several TGFβ responses including senescence and EMT.

## Results

### siSHP2 induces migration

**Fig. 1:**
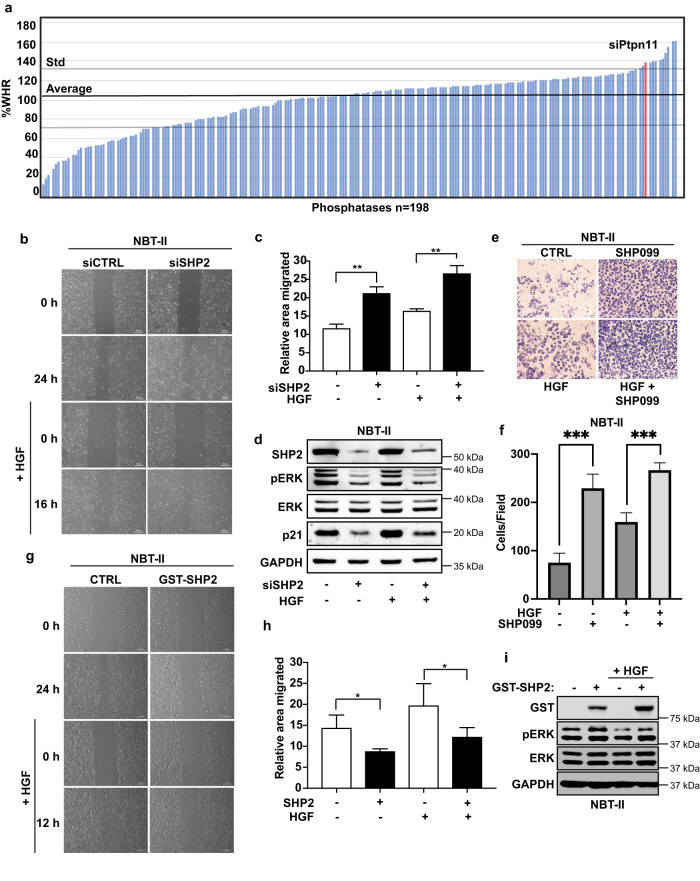
SHP2 regulates cell migration. **a** Bar graph displays percentage wound closure (%WHR) compared to controls obtained from siRNA phosphatase screen. **b** NBT-II cells transduced with siRNA targeting SHP2 or relevant controls were plated for scratch assay and treated with or without HGF (8 μM), panels show migration at 0, 16, and 24 h. Representative images are shown (scale bars, 200 μm). **c** Percentage of migrated area was determined with respect to control (0 h) and a graph was plotted. **P < 0.01 using Student’s *t*-test. Data are mean ± S.D. from three random fields. Data are representative of three independent experiments with similar results. **d** Immunoblot of NBT-II cells from **b** and **c**. Lysates were probed with indicated antibodies. **e** Transwell assay of NBT-II cells treated with SHP099 (5 μM), HGF (8 μM), or in combination for 48 h prior to fixation and crystal violet staining (scale bars 50 μm). **f** Graph represents average number of migrated cells taken from 4 different random fields from (**e**). Data are mean ± S.D. of triplicate samples from a representative experiment performed three times. ***P < 0.001 using Student’s *t*-test. **g** NBT-II cells ectopically expressing GST tagged SHP2 or relevant controls were plated for scratch assay and treated with or without HGF (8 μM), panels show migration at 0, 12, and 24 h. Representative images are shown (scale bars, 200 μm). **h** Percentage of migrated area was determined with respect to control (0 h) and a graph was plotted. *P < 0.05 using Student’s *t*-test. Data are mean ± S.D. from three random fields. Data are representative of three independent experiments with similar results. **i** Immunoblot of NBT-II cells expressing GST-SHP2. Lysates are probed with indicated antibodies.

To gain a further understanding surrounding the mechanisms of hepatocyte growth factor (HGF)-induced migration, we designed a loss-of-function screen to identify phosphatases that alter migration in the bladder cancer cell line NBT-II cells. The phosphatase library consists of pools of siRNAs targeting 198 known phosphatases. NBT-II cells expressing the H2B-mcherry marker were transduced with siRNA targeting each phosphatase in a single-well format. After 48 h, a wound was made using the Incucyte wound maker tool and cells were subsequently treated with or without HGF, a potent inducer of cell migration in NBT-II cells. Wound closure was measured using the Incucyte live cell analysis system with cells imaged every 4 h. Using this stringent protocol, we found that suppression of 55 phosphatases markedly repressed or enhanced cell motility in NBT-II-cells (Fig. 1a and Supplementary Table 1). These 55 genes are implicated in a number of diverse signalling pathways regulating transcription, metabolism, cell trafficking, and cell signalling. Surprisingly, one of the siRNA pools, which significantly enhanced motility compared to controls, targets *PTPN11*, the gene for the phosphatase SHP2. As SHP2 functions downstream of growth factor receptors to upregulate MAPK signalling and pro-proliferative responses, this potentially indicates that loss of SHP2 expression may function through a negative feedback loop to upregulate other key oncogenic pathways involved in migration and proliferation^26^. We therefore focused our attention on SHP2.

We initially sought to validate our screen. We therefore transduced NBT-II cells with siRNAs targeting SHP2 and treated the cells with HGF. As previously observed, SHP2 downregulation significantly enhanced cell motility in both the absence and presence of HGF (Fig. 1b–d). Furthermore, NBT-II cells transduced with siRNAs targeting SHP2 displayed the expected downregulation of phosphorylated ERK (pERK) even in the presence of HGF (Fig. 1d). Similar results were observed in the presence of mitomycin C (Supplementary Fig. 1a–c), once again indicating that the increase in motility is likely independent of increased cellular proliferation.

As SHP2 plays a key role in regulating KRAS and downstream MAPK signalling in cancer, several SHP2 inhibitors have entered clinical trials for the treatment of KRAS mutant tumours. Therefore, we tested if the allosteric SHP2 inhibitor SHP099 displayed similar effects as SHP2 depletion on NBT-II cells and in KRAS mutant lung cancer cells^27^. First, we analysed if SHP099-treated NBT-II cells would alter the invasive capacity of these cells. Indeed, inhibition of SHP2 significantly upregulated invasion (Fig. 1e, f). Next, we tested the effect of SHP099 on KRAS mutant lung cancer cell lines H358 and H1792. In both cases SHP2 inhibition significantly upregulated migration and invasion (Supplementary Figs. 1d, e, 2a, b, d, e). Notably, SHP099 treatment decreased the proliferative capacity of both lung cancer cell lines (Supplementary Fig. 2c, f).

Having established that SHP2 knockdown enhances motility, we tested the effect of SHP2 gain-of-function. In contrast to our knockdown results, overexpression of SHP2 significantly inhibited the migration capacity of NBT-II cells when treated with or without HGF, while expectedly upregulating pERK (Fig. 1g, h, i). Taken together, these results suggest that SHP2 is a negative regulator of cell motility.

### SHP2 regulates TGFβ signalling

**Fig. 2:**
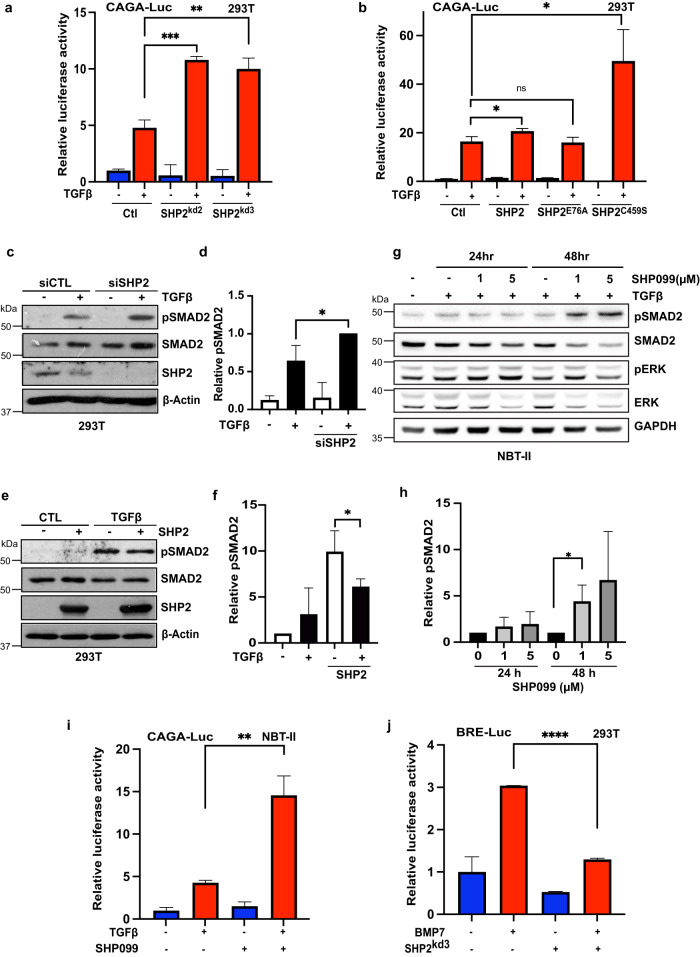
SHP2 regulates TGFβ activity. **a** TGFβ responsive luciferase (CAGA luciferase) of HEK293T cells transfected with two independent hairpins targeting SHP2 or a hairpin targeting GFP were stimulated where indicated with TGFβ (100 pM) overnight before lysis. Error bars represent S.D. of triplicates. Experiments are representative of three independent experiments. **P < 0.01, ***P < 0.001 as determined by Student’s *t*-test. **b** TGFβ responsive luciferase (CAGA luciferase) of HEK293T cells transfected with pcDNA (Ctl), wildtype SHP2, catalytically active SHP2 (E76A), and catalytically inactive mutant of SHP2 (C459S) were stimulated where indicated with TGFβ (100 pM) overnight before lysis. Error bars represent S.D. of triplicates. Experiments are representative of three independent experiments. *P < 0.05 as determined by Student’s *t*-test. **c** HEK293T cells transfected with siRNA targeting SHP2 or relevant control and treated overnight with TGFβ (100 pM). Immunoblotted lysates are probed with indicated antibodies. **d** Quantification of **c** comparing phospho-SMAD2 to corresponding SMAD2. Density was evaluated with IMAGE J. Bars represent mean ± S.D. from three independent experiments. A two-tailed Student’s *t-*test compares the treated populations. *P < 0.05. **e** HEK293T cells transfected with SHP2 or relevant control and treated overnight with TGFβ (100 pM). Immunoblotted lysates were probed with indicated antibodies. **f** Quantification of **e** comparing phospho-SMAD2 to corresponding SMAD2. Density was evaluated with IMAGE J. Bars represent mean ± S.D. from three independent experiments. A two-tailed Students’ *t-*test compares the treated populations. *P < 0.05. **g** NBT-II cells treated with either TGFβ (100 pM) or SHP099 (1 or 5 μM) or both for 24 or 48 h. Lysates were probed with indicated antibodies. **h** Quantification of **g** comparing phospho-SMAD2 to corresponding SMAD2. Density was evaluated with IMAGE J. Bars represent mean ± S.D. from three independent experiments. A two-tailed Student’s *t*-test compares the treated populations. *P < 0.05. **i** TGFβ responsive luciferase (CAGA luciferase) of NBT-II cells stimulated overnight with TGFβ (100 pM) or SHP099 (5 μM) or in combination. Lysates were collected and luciferase measured by a luminometer. Error bars represent S.D. of triplicates. Experiments are representative of three independent experiments. **P < 0.01 as determined by Student’s *t*-test. **j** TGFβ responsive luciferase (CAGA luciferase) of HEK293T cells transfected with shSHP2 or a hairpin targeting GFP were stimulated where indicated with BMP7 (50 μg/μl) overnight before lysis. Error bars represent S.D. of triplicates. Experiments are representative of three independent experiments. ****P < 0.0001 as determined by Student’s *t*-test.

With SHP2 being predominantly recognized as a pro-proliferative factor enhancing MAPK signalling through KRAS activation, it is unlikely that the observed increase in migration following SHP2 inhibition is due to enhanced proliferation as suggested by the mitomycin C results (Supplementary Fig. 1a–c). The TGFβ pathway functions through a number of feedback loops to play a critical role in migration and invasion in numerous cancer models^28^. Therefore, to determine if SHP2 inhibition enhances TGFβ signalling, we co-transfected HEK293T cells with a TGFβ-responsive luciferase reporter (CAGA-Luc) and shRNAs targeting SHP2. Loss of SHP2 expression with two independent shRNA vectors significantly augmented luciferase levels in the presence of TGFβ (Fig. 2a). Next, we sought to address whether the phosphatase activity of SHP2 was essential for TGFβ regulation. The SHP2 protein harbours two tandem SH2 domains, a catalytic protein tyrosine phosphatase (PTP) domain and a C-terminal tail^29^. In its basal state, SHP2 adopts an autoinhibited conformation, in which the first SH2 domain overlaps the catalytic pocket of the PTP domain, thus blocking the active site. Binding of activated proteins to the SH2 domains overcomes autoinhibition. The most frequently observed mutation in SHP2 in cancer is E76K, which dramatically reorganizes the protein exposing the active site of SHP2^30^. Conversely, mutation of SHP2 at C459S within the PTP domain generates a phosphatase null mutant. Ectopic expression of WT SHP2 or the catalytically active SHP2^E76K^ mutant did not appreciably alter luciferase levels compared to controls (Fig. 2b). However, in line with SHP2 knockdown, loss of SHP2 phosphatase activity significantly upregulated CAGA-Luc levels (Fig. 2b). This suggests that SHP2 is a critical regulator of TGFβ activity in TGFβ-responsive cell lines.

R-SMADs are the major conduit regulating the intracellular responses of TGFβ receptor signalling and can be observed as an increase in R-SMAD phosphorylation. In line with the upregulation of CAGA-Luc levels, loss of SHP2 expression intensified SMAD2 phosphorylation, while having no effect on total SMAD2 (Fig. 2c, d). In contrast, ectopic expression of SHP2 slightly decreased the levels of SMAD2 phosphorylation levels (Fig. 2e, f).

Next, we tested if the allosteric SHP2 inhibitor SHP099 displayed similar effects on TGFβ activity^27^. NBT-II cells were treated with either TGFβ ligand or in combination with SHP099. As expected, TGFβ enhanced SMAD2 phosphorylation levels, which was further increased in the presence of SHP099 (Fig. 2g, h). Interestingly, the effect of pSMAD2 upregulation by SHP099 was only observed after 48 h of SHP099 treatment. This discord in pSMAD2 upregulation following SHP2 inhibition was similarly observed in the breast cancer cell lines MDA-MB-436 and BT474, suggesting that the SHP2 effect on TGFβ signalling is nuanced and likely influenced by multiple factors (Supplementary Fig. 3a–d). In accordance with the upregulation of pSMAD2, treatment of NBT-II cells with SHP099 significantly enhanced the effect of TGFβ on the luciferase reporter (Fig. 2i). TGFβ is a member of a large family of structurally related cytokines, including activins, and bone morphogenetic proteins (BMPs). We therefore sought to evaluate if SHP2 inhibition could similarly alter BMP pathway activation. In line with previous results, BMP activation was significantly downregulated in cells depleted for SHP2 (Fig. 2j)^31^. Taken together, these results demonstrate that loss of SHP2 significantly upregulates TGFβ activity. Furthermore, it appears that this effect is specific for TGFβ activation, as we observed contrasting effects on BMP pathway activation.

### SHP2 inhibition activates TGFβ signalling in lung cancer

**Fig. 3:**
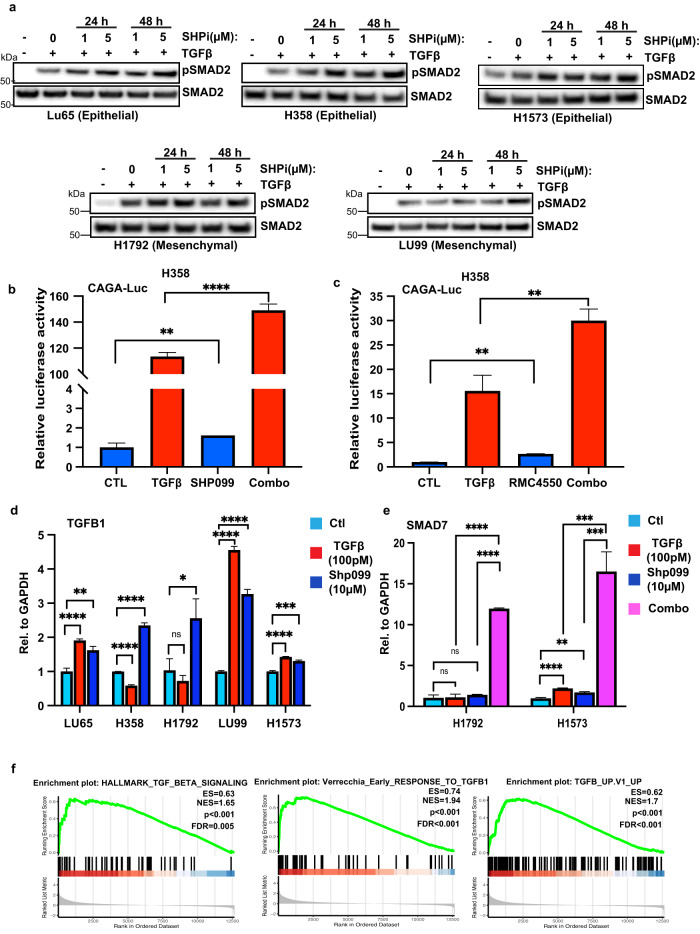
SHP2 inhibition enhances TGFβ activity in KRAS mutant lung cancer. **a** KRAS mutant lung cancer cell lines LU65, H358, H1573, H1792, and LU99 were treated with either TGFβ (100 pM) or SHP099 (1 or 5 μM) or both for 24 or 48 h. Lysates were probed with indicated antibodies. **b** TGFβ responsive luciferase (CAGA luciferase) of H358 cells stimulated overnight with TGFβ (100 pM) or SHP099 (5 μM) or in combination. Lysates were collected and luciferase measured by a luminometer. Error bars represent S.D. of triplicates. Experiments are representative of three independent experiments. **P < 0.01, ****P < 0.0001 as determined by Student’s *t*-test. **c** TGFβ responsive luciferase (CAGA luciferase) of H358 cells stimulated overnight with TGFβ (100 pM) or RMC-4550 (5 μM) or in combination. Lysates were collected and luciferase measured by a luminometer. Error bars represent S.D. of triplicates. Experiments are representative of three independent experiments. **P < 0.01 as determined by Student’s *t*-test. **d** LU65, H358, H1792, LU99 or H1573 cells were stimulated with TGFβ (100 pM) or SHP099 (10 μM) as indicated for 3 h. *TGFB1* mRNA levels relative to *GAPDH* are shown as evaluated by quantitative real time PCR. Data are shown as the mean ± S.D. of triplicate samples from a representative experiment performed two times. *P < 0.05,**P < 0.01, ***P < 0.001, ****P < 0.0001 as determined by Student’s *t*-test. **e** H1792, or H1573 cells were stimulated with TGFβ (100 pM) or SHP099 (10 μM) or in combination as indicated for 3 h. *SMAD7* mRNA levels relative to *GAPDH* are shown as evaluated by quantitative real time PCR. Data are shown as the mean ± S.D. of triplicate samples from a representative experiment performed two times. **P < 0.01, ***P < 0.001, ****P < 0.0001 as determined by Student’s *t*-test. **f** Gene set enrichment analysis of TGFβ (HALLMARK_TGF_BETA_SIGNALLING, VERRECCHIA_EARLY_RESPONSE_TO_TGFB1, TGFB_UP.V1_UP) gene set signatures, extrapolated from the GSE109270 data set derived from five vehicle- and seven SHP099-treated patient derived xenograft tumours. Enrichment scores (ESs), normalized enrichment scores (NESs), *P* values, and false discovery rates (FDRs) are reported.

As SHP2 depletion activated TGFβ signalling in a number of cancer cell lines, we sought to understand the effect of SHP2 inhibition in a KRAS mutant background. SHP2 inhibitors have entered the clinic; however, variable responses have been observed with a number of trials reporting unfavourable toxicity levels halting further studies. Furthermore, several clinical trials have utilised SHP2 inhibitors in combination with the checkpoint inhibitor Nivolumab, a PD-1 antibody. TGFβ is a well-recognized immunosuppressive cytokine and upregulated by multiple cell types in the tumour microenvironment^32^. The lack of response exhibited by checkpoint inhibitors in the clinical setting has been associated with TGFβ signalling in cancer-associated fibroblasts^33^. We, therefore, sought to determine if SHP2 inhibitors enhanced TGFβ signalling in KRAS mutant lung cancer models. In line with our previous results, co-treatment with SHP099 significantly upregulated phosphorylated SMAD2 in all cell lines tested, irrespective of their epithelial or mesenchymal phenotype status (Fig. 3a). No change was observed in the levels of the co-SMAD, SMAD4 (Supplementary Fig. 3e, f). Likewise, co-treatment with SHP099 upregulated the CAGA-Luc reporter compared to TGFβ alone in H358 cells (Fig. 3b). Analysis with a second SHP2 inhibitor, RMC-4550, displayed similar results (Fig. 3c)^34^. Interestingly, inhibition of downstream MEK or AKT decreased overall luciferase levels in both H358 and H1792 cells (Supplementary Fig. 3g, h). In contrast, MEK or AKT inhibition induced TGFβ pathway activation in HEK293T cells and BT474 cells (Supplementary Fig. 3i, and data not shown). Once again, SHP2 depletion decreased BMP pathway activation in H358 cells (Supplementary Fig. 3j).

Next, we analysed the RNA expression levels of the TGFβ target genes *p21, PAI1, SMAD7*, and *TGFB1* following SHP099 treatment in KRAS mutant lung cancer cell lines. In all cell lines tested, SHP2 inhibition consistently upregulated TGFβ-associated gene expression (Fig. 3d, Supplementary Fig. 4a–c). Furthermore, co-treatment of SHP099 and TGFβ significantly upregulated SMAD7 expression compared to either treatment alone (Fig. 3e). To confirm our in vitro results, we stained H358 established xenografts treated with SHP099 for pSMAD2 (Supplementary Fig. 4d, e). Consistent with our data, SHP099 enhanced the levels of pSMAD2, while decreasing the proliferative capacity of lung cancer tumour growth (Supplementary Fig. 4d, e). To further correlate our in vivo data, we probed an RNA data set established from a KRAS mutant patient-derived xenograft (PDX) treated with SHP099. RNA sequencing data was obtained from five vehicle- and seven SHP099-treated PDX tumours^8^. RNA sequence analyses of vehicle and SHP099-treated PDX tumours revealed an increase in the levels of five established “TGFβ signatures” (Fig. 3f, Supplementary Fig. 4f)^35–39^. Collectively, these results suggest that SHP2 inhibition induces TGFβ signalling in KRAS mutant lung cancer models.

### SHP2 dephosphorylates SMURF2

**Fig. 4:**
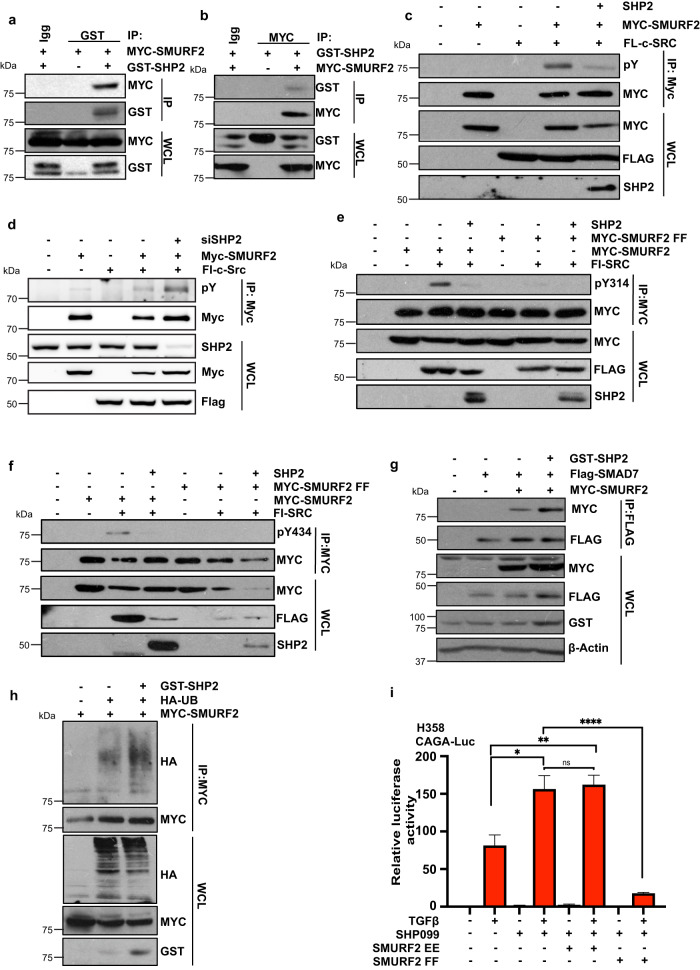
SHP2 binds and dephosphorylates SMURF2. **a** HEK293T cells were transfected with MYC-tagged SMURF2 and/or GST-tagged SHP2. After 48 h, cells were lysed and immunoprecipitated with GST antibody. Immunoprecipitated lysates and whole cell extracts were probed with the indicated antibodies. **b** HEK293T cells were transfected with MYC-tagged SMURF2 and/or GST-tagged SHP2. After 48 h, cells were lysed and immunoprecipitated with MYC antibody. Immunoprecipitated lysates and whole cell extracts were probed with the indicated antibodies. **c** HEK293T cells transduced with SHP2 or relevant controls along with MYC-tagged SMURF2 and FLAG-tagged c-SRC. Lysates were immunoprecipitated with anti-MYC affinity resin. Immunoprecipitated lysates and whole cell extracts were probed with the indicated antibodies. pY signifies tyrosine phosphorylation. **d** HEK293T cells transduced with siRNA targeting SHP2 or relevant controls along with MYC-tagged SMURF2 and FLAG-tagged c-SRC. Lysates were immunoprecipitated with anti-MYC affinity resin. Immunoprecipitated lysates and whole cell extracts were probed with the indicated antibodies. pY signifies tyrosine phosphorylation. **e** HEK293T cells transduced with SHP2, FLAG-tagged c-SRC, and MYC-tagged WT SMURF2, or SMURF2 mutant (SMURF2 Y314F/Y434F, (SMURF2 FF)). Lysates were immunoprecipitated with anti-MYC affinity resin. Immunoprecipitated lysates and whole cell extracts were probed with the indicated antibodies. Y314 phosphorylation was determined with a SMURF2 specific Y314 phospho-antibody. **f** HEK293T cells transduced with SHP2, FLAG-tagged c-SRC, and MYC-tagged WT SMURF2, or SMURF2 mutant (SMURF2 Y314F/Y434F, (SMURF2 FF)). Lysates were immunoprecipitated with anti-MYC affinity resin. Immunoprecipitated lysates and whole cell extracts were probed with the indicated antibodies. Y434 phosphorylation was determined with a SMURF2 specific Y434 phospho-antibody. **g** HEK293T cells were transfected as indicated with MYC-tagged SMURF2, Flag-tagged SMAD7, and/or GST-tagged SHP2. After 48 h cells were lysed and immunoprecipitated with anti-FLAG affinity resin. Immunoprecipitated lysates and whole cell extracts were probed with the indicated antibodies. **h** HEK293T cells transfected with wild-type MYC-SMURF2, HA-tagged ubiquitin and/or GST-SHP2. Following immunoprecipitation of MYC-SMURF2, lysates were resolved by SDS-PAGE and probed with indicated antibodies. **i** TGFβ responsive luciferase (CAGA luciferase) of H358 cells stimulated overnight with TGFβ (100 pM) or SHP099 (10 μM) or in combination with or without ectopic expression of SMURF2 EE or SMURF2 FF. Lysates were collected and luciferase measured by a luminometer. Error bars represent S.D. of triplicates. Experiments are representative of three independent experiments. *P < 0.05, **P < 0.01, ****P < 0.0001 as determined by Student’s *t*-test.

Next, we sought to elucidate the intrinsic mechanism behind the enhanced TGFβ response in SHP2-depleted cells. SMURF2 has previously been demonstrated to act as a bimodal switch between MAPK and TGFβ signalling^26^. Considering that SHP2 has a pivotal role in the activation of the RAS/MAPK pathway and SMURF2 has previously been demonstrated to regulate RAS signalling, we asked whether SHP2 forms a complex with SMURF2 and is involved in the dephosphorylation of SMURF2. Utilising the TGFβ-responsive cell line HEK293T, we performed co-immunoprecipitation assays with GST-tagged SHP2 and MYC-tagged SMURF2. We found that immunoprecipitation of SHP2 from lysates of co-transfected cells resulted in co-precipitation of SMURF2. We also detected this interaction reciprocally by immunoprecipitating MYC-tagged SMURF2 and probing the blotted precipitate with an anti-GST antibody (Fig. 4a, b). The tyrosine kinase c-SRC is a key mediator of SMURF2 activity by phosphorylating tyrosine residues 314 (Y314) and 434 (Y434), resulting in the inhibition of SMURF2 activity and upregulation of TGFβ signalling. To test whether the tyrosine phosphatase SHP2 directly dephosphorylates SMURF2, we co-transfected SMURF2 with c-SRC in the presence or absence of SHP2. As expected, c-SRC enhanced the tyrosine phosphorylation of SMURF2; however, ectopic expression of SHP2 significantly decreased overall tyrosine phosphorylation levels (Fig. 4c). In contrast, SHP2 knockdown cells displayed a significant upregulation of SMURF2 tyrosine phosphorylation (Fig. 4d). Next, we sought to determine if SHP2 dephosphorylated the key phosphorylation residues mediated by c-SRC. Indeed, co-expression of SHP2 completely depleted the phosphorylation of Y314 and Y434 on SMURF2 (Fig. 4e, f). Notably, no SMURF2 phosphorylation was detected in cells expressing a SMURF2 construct, with both tyrosine residues replaced with phenylalanine (SMURF2 FF). SMAD7 forms a complex with SMURF2 blocking the intramolecular interactions within the SMURF2 protein and resulting in enzymatic activation of SMURF2 E3 ligase activity. Specifically, SMAD7 binds to the WW3 domain of SMURF2, which overlaps residue Y314. Phosphorylation of this residue by c-SRC alters the physical nature of this domain, blocking SMAD7 binding^26^. We, therefore, hypothesized that dephosphorylation of Y314 by SHP2 would increase the binding of SMAD7 to SMURF2, resulting in the activation of SMURF2. As depicted in Fig. 4g, co-expression of SHP2 significantly enhanced SMAD7 binding and increased the autoubiquitination of SMURF2, an acknowledged marker of SMURF2 activity (Fig. 4g, h). In line with these results, ectopic expression of the unphosphorylated form of SMURF2, SMURF2 FF, completely annulled the upregulation of TGFβ signalling in H358 cells treated with TGFβ and SHP099 (Fig. 4i). In contrast, co-expression of the phospho-mimetic mutant SMURF2 EE did not further increase luciferase levels compared to SHP099 treatment alone (Fig. 4i). Taken together, these results suggest that SHP2 is a critical regulator of SMURF2 enzymatic activity by dephosphorylating Y314 and Y434 on SMURF2. Dephosphorylation of SMURF2 enhances SMAD7 binding and, consequently, activation of SMURF2. Furthermore, our data suggest that SMURF2 is the primary target of SHP2 and TGFβ pathway activation as SHP2 inhibition did not further alter canonical TGFβ signalling in the presence of SMURF2 phospho-mimetic or phospho-null mutants.

### TGFβ suppression inhibits SHP099-induced senescence

**Fig. 5:**
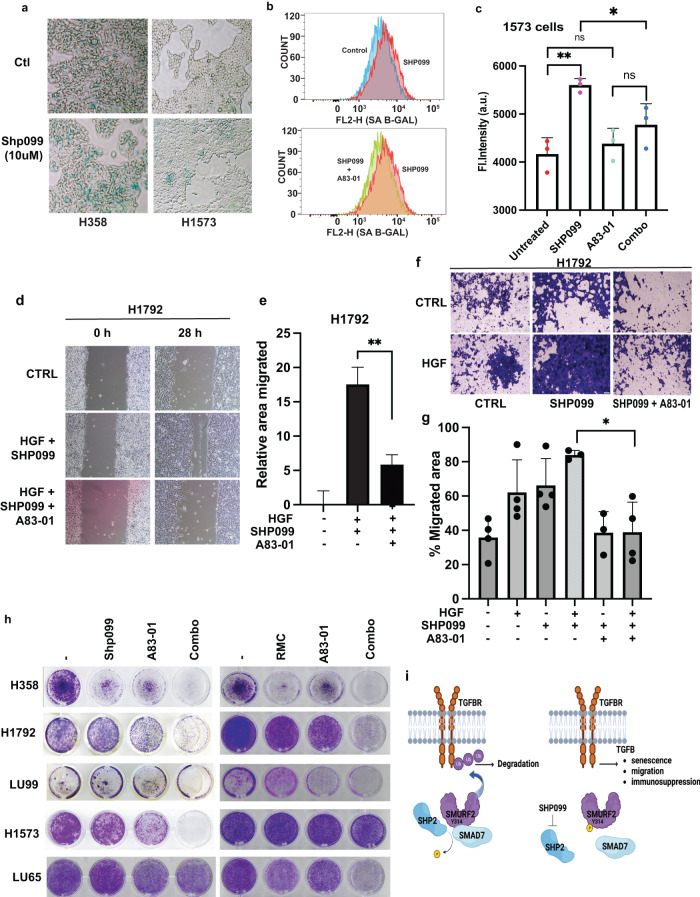
TGFβ inhibition inhibits SHP2-mediated TGFβ responses. **a** SA-β-Gal staining of H358 and H1573 lung cancer cells treated with or without SHP099 for 10 days. Scale bars, 200 μm. The images are representative of two independent experiments. **b**, **c** Representative image of flow cytometry (senescence, **b**) and quantification (**c**) of H1573 cells. After 72 h cells were stained for SA-β-Gal activity. Error bars in C represent S.D. of triplicate experiments. *P < 0.05, **P < 0.01 as determined by Student’s *t*-test. **d** H1792 cells were plated for scratch assay and treated with HGF (8 μM) and SHP099 (10 μM) with or without A83-01 (10 μM), panels show migration at 0 and 28 h. Representative images are shown (scale bars, 200 μm). **e** Percentage of migrated area was determined with respect to control (0 h) and a graph was plotted. **P < 0.01 using Student’s *t*-test. Data are mean ± S.D. from three non-overlapping fields. Data are representative of three independent experiments with similar results. **f** Transwell assay of H1792 cells treated with SHP099 (10 μM), HGF (8 μM), or in combination with or without A83-01(10 μM) for 16 h prior to fixation and crystal violet staining (scale bars 100 μm). **g** Graph represents percent number of migrated cells taken from four different random fields from **f**. Data are mean ± S.D. of triplicate samples from a representative experiment performed three times. *P < 0.05 using Student’s *t*-test. **h** Colony formation assay of H358, H1792, LU99, H1573, and LU65 cells treated with SHP099 (10 μM), RMC-4550 (5 μM), A83-01 (8 μM) or in combination as indicated for 10 days cultured in medium containing 10% FBS. Images are representative of three independent experiments. **i** Schematic overview of SHP2 regulation of SMURF2 and the effect of SHP2 inhibition by SHP099 on TGFβ pathway activation. Figure was generated using Biorender.com.

SHP2 inhibition induces a senescence-like phenotype in lung cancer models but only in reduced serum conditions^8^. Similarly, the activation of the TGFβ pathway triggers cellular senescence^40^. However, in several cellular contexts, prolonged TGFβ exposure mediated by autocrine effects is required prior to observing any perceptible phenotypic changes^41^. As SHP2 inhibition induces TGFβ, we speculated that loss of TGFβ signalling may inhibit SHP2-induced senescence and render cells sensitive to SHP2 loss. Consistent with previous results, we observed no significant increase in SA-β-gal staining in high serum conditions (data not shown)^8^. However, we previously demonstrated that TGFβ activity is profoundly induced in all cell lines tested in 10% serum. We therefore sought to analyse the effects of SHP099 on changes in cell morphology and senescence after prolonged treatment. Lung cancer cell lines were treated with SHP099 for 10 days and either stained directly with SA-β-gal or collected and FACS-sorted for SA-β-gal-positive cells. Only the epithelial-like cell lines H358 and H1573 demonstrated a significant increase in SA-β-gal staining (Fig. 5a and data not shown). Interestingly, co-treatment with the TGFβ receptor inhibitor A83-01 significantly decreased SA-β-gal staining in both of these cell lines (Fig. 5b, c, Supplementary Fig. 5a). Next, we sought to assess the effect of TGFβ inhibition on SHP099-induced migration and invasion in KRAS mutant lung cancer models. As expected, downregulation of TGFβ activity completely annulled SHP099-induced migration and invasion in both H1792 and H358 cells (Fig. 5d–g, Supplementary Fig. 5b–d). As downregulation of SHP2 enhanced TGFβ activity, we investigated whether inhibition of TGFβ would perturb migration in NBT-II depleted for SHP2 and treated with HGF. We observed that co-treatment with the TGFβ receptor inhibitor A83-01 completely blocked cellular migration in both control cells and cells with downregulation of SHP2 (Supplementary Fig. 5e, f). Similarly, TGFβ inhibition decreased the invasive capacity of these cell lines induced by SHP099 treatment (Supplementary Fig. 5g, h). Next, we sought to understand what effect TGFβ suppression would have on overall cell survival on cells treated with SHP099 or RMC-4550. Although all cell lines displayed some reduction in overall cell proliferation with the two SHP2-targeting compounds, only H358 cells displayed a consequential reduction (Fig. 5h, Supplementary Fig. 6a, b). Treatment of lung cancer cell lines with the TGFβ inhibitor significantly downregulated the proliferative capacity of all cell lines; however, partial resistance to A83-01 monotherapy was observed. Importantly, co-treatment of SHP2 inhibitors and TGFβ receptor inhibitor significantly blocked proliferation compared to either treatment alone. This anti-proliferative effect was observed in all cell lines but most notably in the epithelial-like cell line H358, and the mesenchymal-like cell lines H1792, and LU99 (Fig. 5h, Supplementary Fig. 6a, b). Next, we sought to investigate whether the decrease in cell proliferation in cells treated with the combination of SHP2 and TGFβ inhibitors was a result of growth arrest or an increase in apoptosis. Cell cycle analysis of H1573 cells by FACS indicated that SHP099 treatment induced a significant increase in G1 but little to no apoptosis, as determined by an accumulation of cells in Sub-G1. However, the co-addition of A83-01 only slightly increased the Sub-G1 population without significantly altering the cell cycle profile compared to SHP099 treatment alone, suggesting that TGFβ inhibition affects proliferation or survival independent of cell cycle progression in this cell line (Supplementary Fig. 6c). Collectively, our data suggest that the combination of SHP2 and TGFβ inhibitors is effective at reversing SHP2 inhibitor-induced senescence and decreasing survival in KRAS mutant lung cancer models, likely independent of apoptotic mechanisms.

## Discussion

Targeted therapies have demonstrated inconsistent effects as anti-cancer agents when administered to patients with well-defined tumour-driving lesions due to compensatory mechanisms and feedback loops. However, targeting common downstream nodes shared by multiple RTKs signalling pathways has emerged as a promising strategy. SHP2 acts as a convergent node downstream of multiple RTKs and plays a crucial role in regulating RAS activation. While RTKs typically activate RAS through the GRB2-SOS1 complex without relying on SHP2, the proliferation of KRAS (G12C) mutant cancer cells in vivo necessitates SHP2 activity. Additionally, reactivation of KRAS following inhibition is mediated through a SHP2-dependent feedback loop. Therefore, inhibiting SHP2 has emerged as a clinical target by effectively suppressing RAS-mediated signalling and overcoming adaptive resistance. Overall, however, clinical success for SHP2 has been greatly limited by their narrow therapeutic window and adverse effects, indicating that a SHP2 inhibitor is unlikely to work on its own^42^. Promising preclinical results have been obtained by combining SHP2 inhibitors with KRAS (G12C) or other pathway-specific inhibitors, leading to significant reductions in tumour volume^42^. Furthermore, several clinical trials are underway for several SHP2 inhibitors, such as JAB-3068, RLY-1971, SHP099 and TNO155, either as monotherapy or in combination with KRAS (G12C) inhibitors, EGFR inhibitors, SOS-1 or RAF inhibitors, demonstrating the potential of targeting SHP2 to combat adaptive reactivation of KRAS in cancer (NCT04330664, NCT04185883, NCT04699188, NCT04973163, and NCT04975256). Importantly, SHP2 inhibition may also yield immunomodulatory effects in T cells and macrophages to elicit antitumor immune responses. The inhibitory receptor PD-1 blocks T cell activation through a process attributed to the recruitment of the phosphatase SHP2 to its cytoplasmic tail^43^. Because of this, it is expected that deletion of SHP2 would abrogate the inhibitory pathway activated downstream of PD-1 receptor enhancing the effect of checkpoint inhibitors.

The role of TGFβ in cancer is complex and nuanced with TGF-β functioning as both a tumour suppressor or, conversely, as an oncogene. Importantly, TGFβ has been demonstrated to induce resistance to multiple targeted therapies likely by inducing changes in cell plasticity leading to various states of drug tolerance^44,45^. Furthermore, TGFβ exerts systemic immune suppression and inhibits host immunosurveillance^46^. Recently, it has been demonstrated that a lack of response to anti-PD-L1 is associated with high levels of TGFβ and can be circumvented with the use of TGFβ blocking antibodies^33^. Using a functional RNAi screen targeting all the known phosphatases, we now identify SHP2 in the regulation of TGFβ signalling. We demonstrate that in all cell lines tested, SHP2 inhibition by genetic or chemical means induces TGF-β signalling, resulting in enhanced TGF-β-mediated responses including senescence, migration, invasion, and survival.

In this setting, SHP2 dephosphorylates two key residues on the E3 ligase SMURF2 Y314 and Y434 permitting binding of the scaffold protein SMAD7 and interrupting the intramolecular interactions associated with enzymatic inactivation of the protein. This permits SMURF2 to ubiquitinate the TGFβ receptor complex targeting it for proteosome-mediated deubiquitination and downregulation of TGFβ pathway activation (Fig. 5i). Conversely, inhibition of SHP2 maintains the phosphorylation of both of these sites inhibiting SMURF2 activity and maintaining TGFβ activity (Fig. 5i). We have previously demonstrated that these two sites are directly phosphorylated by the tyrosine kinase c-SRC. Similarly, c-SRC-mediated tyrosine phosphorylation of KRAS and RUNX1 negatively affects KRAS and RUNX1 activity. In all three cases SHP2 dephosphorylation rescued the negative regulation by SRC^47,48^. In addition, SHP2 plays a signalling role by dephosphorylating other molecules, including negative regulators of SPROUTY and activators of SRC, through the dephosphorylation of SRC-regulatory proteins^49^. SHP2 inhibition has also been demonstrated to upregulate TGF-β signalling in Prrx1-expressing mesenchymal progenitors resulting in severe defects in calvarial bone formation^31^.

Our data clearly indicates that SHP2 depletion induces TGFβ associated phenotypes including migration and invasion in bladder and lung cancer models and senescence. These effects could be circumvented by the co-treatment with TGFβ inhibitors. TGFβ is known to induce a number of cytostatic effects by mediating the expression of a number of cyclin-dependent kinase inhibitors including p15^Ink4b^, p21, and p27. This suggests a senescence promoting role of TGFβ under normal conditions and also coincides with the tumour suppressing role of cell senescence. Furthermore, TGFβ pathway activation in tumours can drive immune evasion and chemotherapy resistance as a consequence of EMT induction and carcinoma cell plasticity^50^. Our analysis of RNA sequencing data derived from SHP099-treated PDX tumours ordered by normalized enrichment score ranked the HALLMARK-EMT and FOROUTAN_TGFB_EMT_UP signatures as the most significant (Supplementary Fig. 7A). It is therefore likely that SHP2 inhibition induces an array of TGFβ mediated phenotypes based on the genetic background of the tumour. This may also partially explain why treatment with TGF-β inhibitors in combination with SHP2 inhibitors significantly decreased the proliferation capacity of multiple cell lines even though SHP2 depletion did not induce senescence in all cell lines tested. We have previously demonstrated that TGFβ inhibition induces MAPK kinase activation through a hereto unknown mechanism indicating that downregulation of MAPK-mediated proliferation by SHP2 inhibitors and TGFβ-mediated changes in cellular plasticity by TGFβ inhibition is effectively required to block cellular proliferation^26^. As cells cannot proliferate and undergo EMT at the same time, it is tempting to speculate that a quasi-senescent state is required for cells to undergo the appropriate transcriptional changes to allow these cellular changes to occur^51^. Recent evidence has indicated that a senescence-associated secretory phenotype can contribute to tumour progression by potentially playing a role in EMT^52^. Although the pathways involved in senescence and EMT are fundamentally different, we must continue to build on our current understanding of the effects of targeted therapies on the tumour microenvironment, which will aid in identifying clinically relevant combinations, in particular those associated with immunotherapies.

Collectively, our findings identify SHP2 as a regulator of SMURF2 and TGFβ signalling. Furthermore, we demonstrate that SHP2 inhibition potently leads to the induction of TGFβ, suggesting that combination therapy with SHP2 inhibitors and TGFβ inhibitors should be considered in lung cancer patients with activated KRAS.

## Methods

### Screening materials methods

Stably transfected NBT-II H2B-mcherry cells were seeded at a density 5 × 10^5^ cells/ml in each well of a Incucyte Imagelock 96 well plate (Essen Bioscience) and transfected with 20 nM siRNA (Qiagen) for 48 h. Wounds were made for each well using the Incucyte 96-well wound maker tool (Essen Bioscience). Each well was washed twice with PBS before replenishing with cell culture media with or without 5 ng/ml HGF (R&D Systems) and the plate was placed into the Incucyte Live Cell Analysis System and imaged every 4 h for a duration of 24 h. Wound closure and proliferation rates were measured using the Incucyte Scratch Wound Analysis software.

### Western blotting and quantification

Cells were lysed in solubilizing buffer (50 mM Tris pH 8.0, 150 mM NaCl, 1% NP-40, 0.5% deoxycholic acid, 0.1% SDS, 200 µM Sodium Vanadate, 1 µM magnesium chloride, 50 mM sodium fluoride, 25 mM β-glycerol phosphate), supplemented with protease inhibitors (Complete; Roche). Whole cell extracts were then separated on 7–12% SDS-Page gels and transferred to polyvinylidene difluoride membranes (Millipore). Before antibody probing, membranes were blocked with bovine serum albumin except when antibody probing was for phospho-SMAD2, in which case the membrane was blocked in milk. Blots were then incubated with an HRP-linked secondary antibody and the signal was detected with chemiluminescence (Pierce) using film and developed using film developer (Konica Minolta). All original images are provided (Supplementary Fig. 8). All blots within each relevant panel were derived from same experiment and processed in parallel. Image-J software (https://imagej.nih.gov/ij/) was used to quantify resultant Western blots.

### Scratch wound assay

Cells were seeded (300,000 cells per well) in 6-well plates. Either transfection with siSHP2 or SHP2 overexpression was performed the next day. 48 h after seeding, cells were serum starved with DMEM without FBS for 24 h. Cells were then treated with mitomycin C (5 μg/mL) or DMSO for 2 h. A scratch was created with a sterile p10 pipette tip and the cells were washed with 1X PBS. Then, the medium was replaced with DMEM containing 1% FBS in the presence or absence of 5 ng/mL HGF. Images were taken immediately for the 0-h time point. The plate was then returned to the incubator and imaged again at various time points. At least four images were taken per condition. To quantify the area migrated, each image was sectioned into 10 equal columns. A visual scoring between 0 to 10 was given for each column based on the confluency (with 0 being no cells and 10 being confluent). The total score for each image was the sum of all 10 columns. The average total score of the 0-h time point was subtracted from the total score of images from the various time points to determine the relative area migrated.

### Transwell migration assay

Cells were grown in medium supplemented with 10% FBS in 10 cm dishes to 80% confluency. The cells were then serum-starved for 24 h. After 24-hour starvation, the cells were treated with SB-431542 (5μM) or A83-01(10 μM) with or without SHP099 (10 μM) or DMSO for 2 h. After 2 hours, 5 × 10^4^ cells were suspended in serum-free medium containing relevant drugs or DMSO and seeded on the upper compartments of the cell culture inserts (BD Falcon, 8μm pore, transparent polyethylene terephthalate (PET) track-etched membrane; BD Biosciences, Heidelberg, Germany). The lower chambers were filled with 10% FBS supplemented medium, in the presence or absence of HGF (5 ng/mL). After 48 h, excess media and non-migrated cells were removed from the upper compartment of the insert using a cotton-tipped swab. The migrated cells were fixed in 70% ethanol for 10 min and stained with crystal violet solution. Migrated cells were then visualised through brightfield microscope and pictures were taken at four random sites and quantified.

### Plasmids and antibodies

The following plasmids were purchased from Addgene: MYC-SMURF2 (#13678), MYC-SMURF2 C716A (#13678), MYC-SMURF2 FF29/30AA (#24604) and HA-Ubiquitin (#17608). FLAG-SMAD7 was a kind gift from Joan Seoane. CAGA luciferase and SV40-Renilla were kind gifts from Rene Bernards. pLKO1-SHP2-1 5′GCAGTTAAATTGTGCGCTGTA3′, pLKO1-SHP2-1 5′ CGCTAAGAGAACTTAAACT TT 3’. Additional cloning information will be given upon request. The following antibodies were purchased from Cell Signalling Technologies: anti-p-SMAD2 (#3101), anti-SMAD2 (#3103), anti-pERK (#4370), anti-ERK (#4695), anti-SMAD4 (#38454), anti-GST (#2622), and anti-SHP2 (#3397). The following antibodies were purchased form Santa Cruz Biotechnology: anti-HA (#sc805 or #sc57592), anti-pY(#sc7020), anti-p21 (#sc471), anti-GAPDH(#sc32233), and anti-MYC (#sc40 or #sc78). The following antibodies were purchased from Sigma-Aldrich: anti-FLAG (#F7425) and anti-β-ACTIN (#A1978). Phospho-specific antibodies to Tyr314 and Tyr434 of SMURF2 were generated by Biomatik, USA. Rabbit polyclonal antibodies were raised against specific peptide sequences. For Tyr314, peptide sequence corresponding to CEIRNTATGRV(pY)FVDHN was used to raise antibodies against the phosphorylated form and CEIRNTATGRVYFVDHN for the unphosphorylated form. Similarly, for Tyr434, peptide sequence corresponding to CLWKRLMIKFRGEEGLD(pY)GGVAR was used to raise antibodies against the phosphorylated form and CLWKRLMIKFRGEEGLDYGGVAR for the unphosphorylated form. The antibodies were received in lyophilized form, which was further dissolved using ddH2O, except antibodies for the unphosphorylated Tyr434, which could only be dissolved in 10% DMSO. All antibodies were used at a dilution of 1:1000.

### Cell culture and transient transfections

HEK293T and NBT-II cells were cultured in Dulbecco’s modified Eagle medium (DMEM- High glucose with L-glutamine (Hyclone)) supplemented with 10% fetal bovine serum (Hyclone), 1% sodium pyruvate (Hyclone) and 1% Penicillin/Streptomycin (Gibco). Lung cancer cell lines H358, H1573, LU99, LU65, H1792 were cultured in RPMI with L-glutamine supplemented with 10% fetal bovine serum (Hyclone). MDA-MB-436 and BT474 were maintained in DMEM supplemented with 10% fetal bovine serum, 2 mM L-glutamine, 1% Penicillin/Streptomycin. All cell lines were acquired from ATCC and regularly tested for mycoplasma contamination by PCR. HEK293T cells were divided in 10-cm dishes 1 day prior to transfection. Sub-confluent cells were transfected using the calcium phosphate transfection method^53^. Cells were incubated overnight and washed twice in PBS. Lysates were collected 48–72 h post transfection. When appropriate, TGFβ (100 pm; R&D), A83-01 (8 µM: Selleck), SHP099 (10 µM, Selleck), RMC-4550 (10 µM, Selleck), MG132 (10 µM; Calbiochem) MEK162 (1 µM, Selleck), GDC-0068 (1 µM, Selleck) were added. For cell number quantification cells were incubated on the OLYMPUS Provi CM20 and resulting data was analysed using the associated software.

### Luciferase assays

Luciferase assays were performed in a 12-well plate using the Dual luciferase system (Promega). CAGA-luciferase vector well (200 ng per well) and SV40-Renilla (40 ng per well) was transfected in the presence of SHP2 WT (400 ng per well), or either SHP2 mutants (400 ng per well), or a control vector. For loss-of-function experiments, CAGA-luciferase vector (200 ng per well) and SV40-Renilla (40 ng per well) was co-transfected with 1.5 μg per well of relevant siCTL control vector or siSHP2 knockdown vectors or 0.5 μg per well SMURF2 mutants. After 72 h 100 pM TGFβ was added in the presence of DMEM (0% FCS) and luciferase counts were measured approximately 16 h later using a Sirius Luminometer (Berthold).

### Immunoprecipitation and In vivo deubiquitination assay

For coimmunoprecipitation experiments cells were lysed in ELB (0.25 M NaCl, 0.5% NP-40, 50 mM HEPES [pH 7.3]) supplemented with proteasome inhibitors (Complete; Roche). Cell lysates (500 μg to 1 mg) were incubated overnight with 1 μg of the indicated antibodies conjugated. Subsequently the lysates were then incubated for up to 6 h with protein A or protein G sepharose beads (GE Healthcare), washed three times in ELB buffer and separated out on SDS-PAGE gels. For in vivo ubiquitination experiments, MYC-SMURF2 (5 μg) was co transfected with HA-Ubiquitin (5 μg) and GST-SHP2 WT (5 μg), or a control vector. After 72 h MG132 (5 μM) was added, incubated overnight, and cells were lysed in ELB buffer.

### Quantitative real-time PCR

Cells were collected, washed twice in PBS and RNA was isolated using GeneJet RNA extraction kit (Thermo-Scientific) and cDNA was synthesized using EvoScript Universal cDNA Master (Roche). qRT was performed using specific mRNA primers (Integrated DNA Technologies) and GoTaq qPCR Master Mix (Promega). Reactions were carried out on Verit 96-well fast thermal cycler or Viia 7 (Applied Biosystems). Relative mRNA values are calculated by the ∆∆Ct method. GAPDH was used as internal normalization controls where specified. The following qPCR primers were used SMAD7: 5′‐AAA CAG GGG GAA CGA ATT ATC‐3′, 5′‐ACC ACG CAC CAG TGT GAC‐3′; GAPDH: 5′‐AAC AGC GAC ACC CAC TCC TC‐3′, 5′‐CAT ACC AGG AAA TGA GCT TGA C‐3′; PAI: 5′‐ AAG GCA CCT CTG AGA ACT TCA‐3′, 5′‐CCC AGG ACT AGG CAG GTG‐3′; p21: 5′‐ CCG AAG TCA GTT CCT TGT GG‐3′, 5′‐CAT GGG TTC TGA CGG ACA T‐3′; TGFB1: 5′‐GCAGCACGTGGAGCTGTA‐3′, 5′‐CAGCCGGTTGCTGAGGTA‐3′.

### SA-β-Gal staining

Lung cancer cell lines were treated for 8–10 days with SHP099 (10 µM, Selleck). Cells were then seeded 50,000 cells in a 6 well plate as follows: control (DMSO-never treated) or SHP099 treated cells (maintained in SHP099). Following 24 h incubation, cells were either treated with DMSO (never treated), SHP099 (SHP099-10 days), A83-01 (SHP099-10 days), or the combination (SHP099-10 days). After 72 h cells, were fixed and stained with cell staining working solution according to the manufacturer’s protocol (Sigma-GALS). For fluorescent detection of Beta Galactosidase (Abcam ab228562), cells were treated as above and stained using the manufacturers protocols. Quantitative analysis was performed in a FACScalibur cytometer using the Cell Quest software and FlowJo.

### Cell viability and Sub-G1 assays

Lung cancer cell lines were seeded in 12 well plates (2 × 10^4^). After 24 h, cells were treated with DMSO, SHP099 (10 µM), RMC-4550 (10 µM), A83-01 (8 µM), or a combination of SHP099/A83-01 or RMC-4550/A83-01 until control wells were full (7–10 days). Cells were washed twice with PBS and fixed with methanol and acetic acid (3:1). After 30 min, cells were washed twice with water, and 1 ml of Coomassie stain (0.1% Coomassie, 50% methanol, and 10% acetic acid) were added. After 30 min cells were washed 3 times in water and air-dried. Cell-cycle and hypodiploid apoptotic cells were quantified by flow cytometry as described in ref. ^54^. Briefly, cells were washed two times in PBS, fixed in 70% cold ethanol and stained with propidium iodide in the presence of RNase. Quantitation analyses of Sub-G1 cells was performed in a FACScalibur cytometer.

### Gene set enrichment analysis

The processed RNA-seq expression of GSE109270 data set was downloaded, and gene set enrichment analysis was performed using gene set enrichment software (GSEA v4.3.2). The HALLMARK_TGF_BETA_SIGNALLING, VERRECCHIA_EARLY_RESPONSE_TO_ TGFB1, TGFB_UP.V1_UP, PLASARI_TGFB1_TARGETS_10HR_UP, REACTOME_TGF_ BETA_SIGNALLING_ACTIVATES_SMADS, FOUROUTAN_TGFB_EMT_UP, AND HALLMARK_EPITHELIAL_MESENCHYMAL_TRANSITION gene sets were used to assess the enrichment of TGFβ -associated gene expression in SHP099 treated patient derived organoids versus vehicle group.

### Xenograft experiments

Suspension of 5 × 10^6^ cells was injected subcutaneously into the flanks of 6- to 8-week-old male nude mice (Chubu Kagaku Shizai Co.,Ltd., Aichi, Japan) under anaesthesia (medetomidine hydrochloride (0.75 mg/kg), midazolam (4 mg/kg), butorphanol tartrate (5 mg/kg)). The care and treatment of experimental animals were in accordance with institutional guidelines. Tumours were randomized (*n* = 6) once the mean tumour volume reached approximately 150–200 mm^3^. Drugs were administered once daily by oral gavage. SHP099 was dissolved in 5% DMSO, 0.5% methylcellulose, and 0.1% Tween 80. Trametinib was dissolved in 0.5% methylcellulose and 1% Tween 80. Mice were monitored daily for body weight and general condition. Tumours were measured twice weekly using calipers, and volume was calculated using the following formula: volume (mm^3^) = length × width^2^ × 0.52. According to institutional guidelines, mice were sacrificed when the tumours they harboured reached a volume of 1000 mm^3^. After euthanasia, tissue samples were immediately collected, snap-frozen in liquid nitrogen, and stored at −80 °C for further analysis. All animal experiments were performed according to the protocols approved by the Institutional Animal Care and Use Committee at Aichi Cancer Centre.

### Supplementary information

Supplementary Information

## Data Availability

Qualified researchers may request materials and/or methods directly from P.J.A.E.
