# Supplementary material for: SHP2 inhibitors maintain TGFβ signalling through SMURF2 inhibition
Source: NPJ Precis Oncol. 2023 Dec 15;7:136. doi: 10.1038/s41698-023-00486-6 (PMC10724235; doi:10.1038/s41698-023-00486-6)
Supplement: Supplementary file 1 — Supplementary Information [file 41698_2023_486_MOESM1_ESM.pdf]

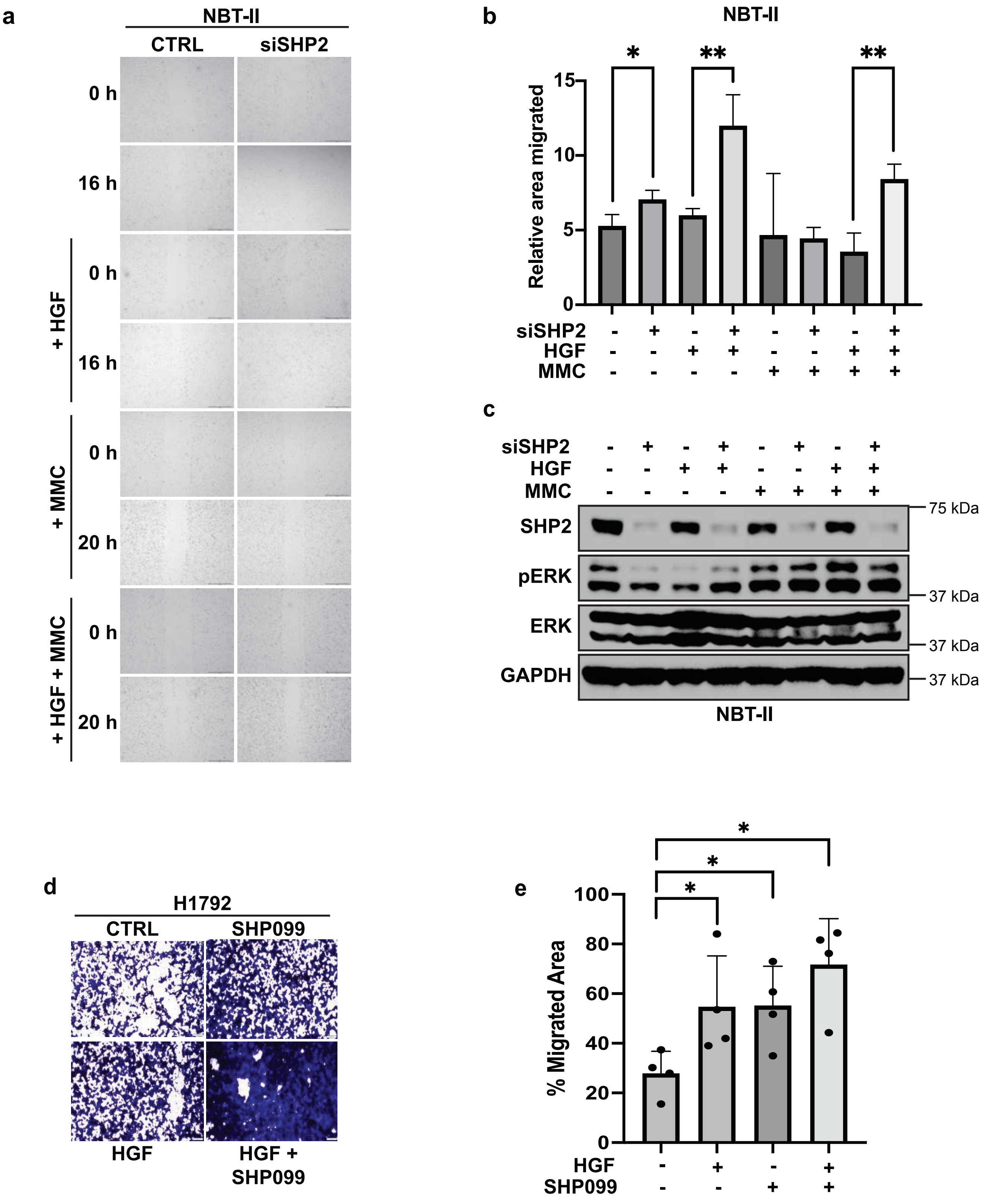

Supplementary Figure 1. SHP2 inhibition enhances invasion in bladder and lung cancer cell lines.

(**a**) NBT-II cells transfected with siRNA targeting SHP2 or relevant controls were plated for scratch assay and treated with either DMSO, HGF (8 μM), mitomycin C (5μg/ml) or HGF (8 μM) in combination with mitomycin C (5μg/ml), panels show migration at 0, 16, or 20 hours. Representative images are shown (scale bars, 650 μm). (**b**). Percentage of migrated area was determined with respect to control (0 h) and a graph was plotted. *P <0.05, **P <0.01 using Student's t-test. Data are mean SD from three random fields. Data are representative of three independent experiments with similar results. (**c**) Immunoblot of NBT-II cells from A and B. Lysates are probed with indicated antibodies. (**d**) Transwell assay of H1792 cells treated with SHP099 (10 μM), HGF (8 μM), or in combination for 16 hours prior to fixation and crystal violet staining (scale bars 100 μm). (**e**) Graph represents percent number of migrated cells taken from four different random fields from panel (d). Data are mean ± SD of triplicate samples from a representative experiment performed three times. *P≤0.05 using Student's t-test.

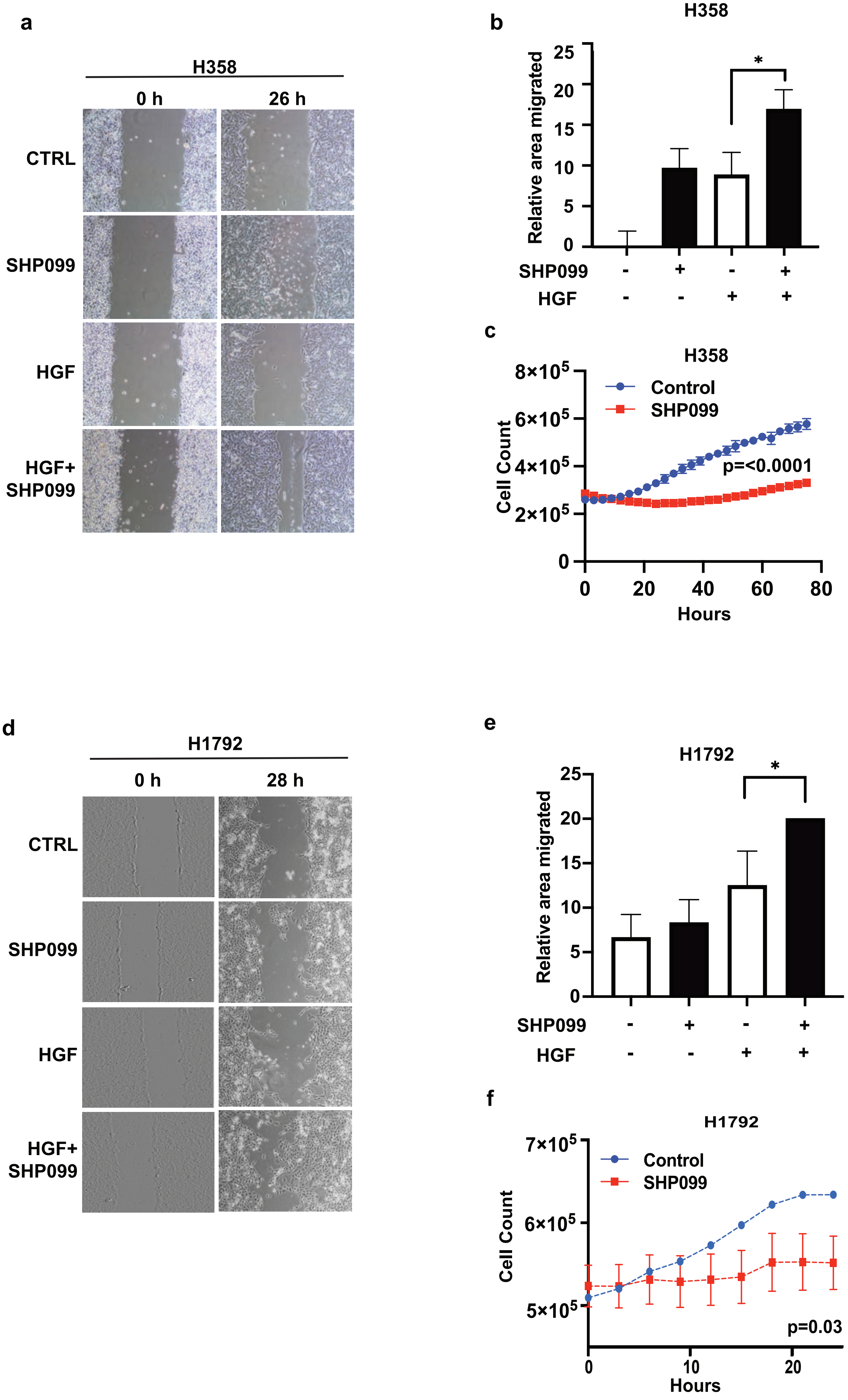

a
H358
0 h
26 h
CTRL
SHP099
HGF
HGF+
SHP099
b
H358
Relative area migrated
SHP099
HGF
c
H358
Control
SHP099
p=<0.0001
Cell Count
Hours
d
H1792
0 h
28 h
CTRL
SHP099
HGF
HGF+
SHP099
e
H1792
Relative area migrated
SHP099
HGF
f
H1792
Control
SHP099
Cell Count
p=0.03
Hours

Supplementary Figure 2. SHP2 inhibition enhances invasion in KRAS mutant lung cancer cell lines.

(**a**) H358 cells treated with SHP099 (10 μM) or DMSO were plated for scratch assay and treated with or without HGF (8 μM), panels show migration at 0 and 26 h. Representative images are shown (scale bars, 200 μm). (**b**) Percentage of migrated area was determined with respect to control (0 h) and a graph was plotted. *P ≤0.05 using Student's t-test. Data are mean ± SD from three random fields. Data are representative of three independent experiments with similar results. (**c**) Overall cell count of H358 cells from A and B treated with or without SHP099 (10 μM) for indicated time points and relevant data was plotted. P ≤0.0001 using Student's t-test. Data are mean ± SD from nine random fields. Data are representative of two independent experiments with similar results. (**d**) H1792 cells treated with SHP099 (10 μM) or DMSO were plated for scratch assay and treated with or without HGF (8 μM), panels show migration at 0 and 28 h. Representative images are shown (scale bars, 200 μm). (**e**) Percentage of migrated area was determined with respect to control (0 h) and a graph was plotted. *P ≤0.05 using Student's t-test. Data are mean ± SD from three random fields. Data are representative of three independent experiments with similar results. (**f**) Overall cell count of H1792 cells from D and E treated with or without SHP099 (10 μM) for indicated time points and relevant data was plotted. P=0.03 using Student's t-test. Data are mean ± SD from nine random fields. Data are representative of two independent experiments with similar results.

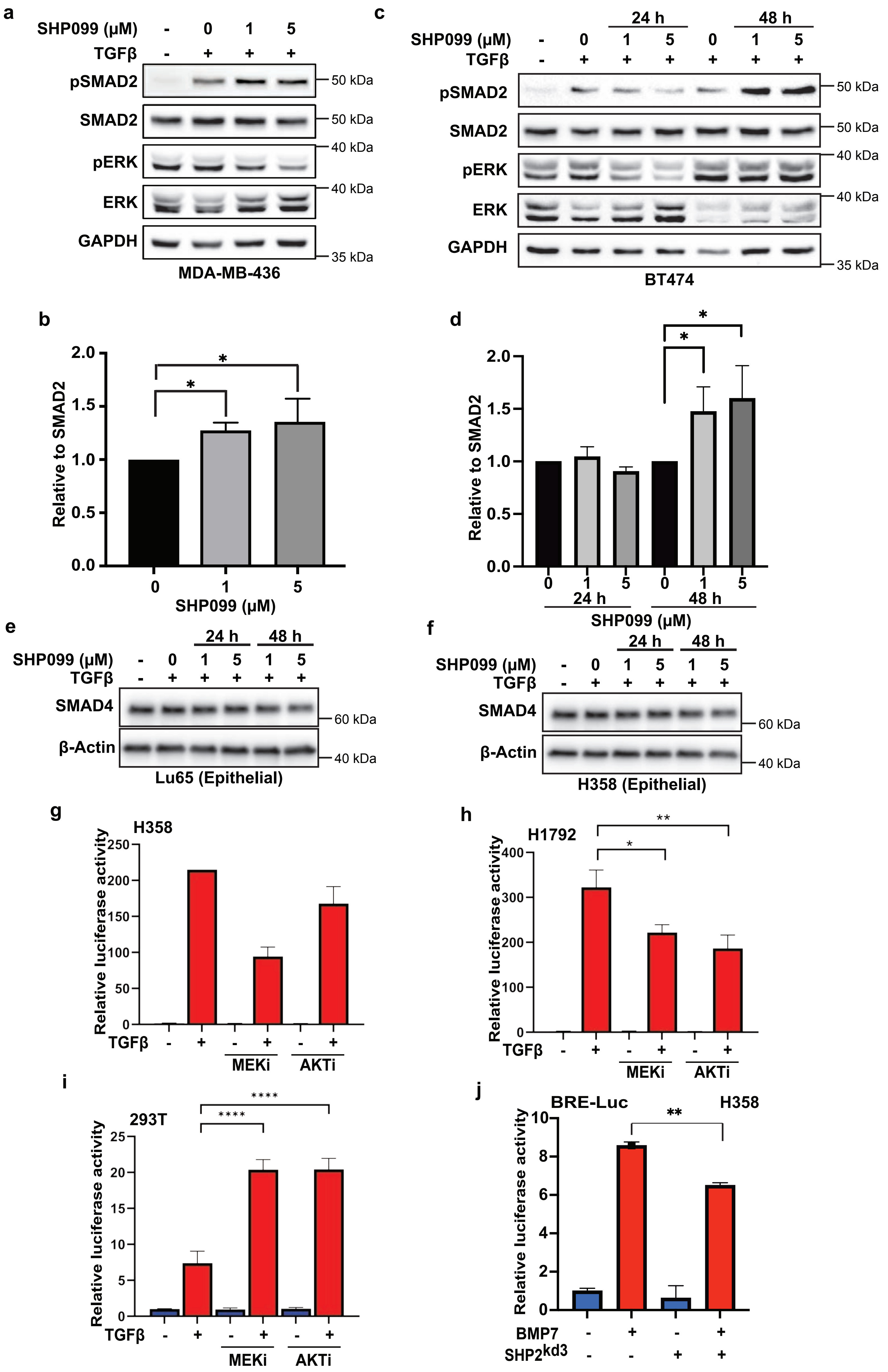

a
SHP099 (μM) - 0 1 5
TGFβ - + + +
pSMAD2
SMAD2
pERK
ERK
GAPDH
50 kDa
50 kDa
40 kDa
40 kDa
35 kDa
MDA-MB-436
c
24 h
48 h
SHP099 (μM) - 0 1 5 0 1 5
TGFβ - + + + + + +
pSMAD2
SMAD2
pERK
ERK
GAPDH
50 kDa
50 kDa
40 kDa
40 kDa
35 kDa
BT474
b
Relative to SMAD2
SHP099 (μM)
d
Relative to SMAD2
24 h
48 h
SHP099 (μM)
e
24 h
48 h
SHP099 (μM) - 0 1 5 1 5
TGFβ - + + + + +
SMAD4
β-Actin
60 kDa
40 kDa
Lu65 (Epithelial)
f
24 h
48 h
SHP099 (μM) - 0 1 5 1 5
TGFβ - + + + + +
SMAD4
β-Actin
60 kDa
40 kDa
H358 (Epithelial)
g
H358
Relative luciferase activity
TGFβ - + - + - +
MEKi
AKTi
h
H1792
Relative luciferase activity
TGFβ - + - + - +
MEKi
AKTi
i
293T
Relative luciferase activity
TGFβ - + - + - +
MEKi
AKTi
j
BRE-Luc
H358
Relative luciferase activity
BMP7 - + - +
SHP2kd3 - - + +

Supplementary Figure 3. SHP2 inhibition activates SMAD2 in breast cancer cell lines.

(**a**) MDA-MB-436 cells treated with either TGFβ (100 pM) or SHP099 (1 or 5 μM) for 24 hours. Lystates were probed with indicated antibodies. (**b**) Quantification of panel A comparing phospho-SMAD2 to corresponding SMAD2. Density was evaluated with IMAGE J. Bars represent mean ± SD from three independent experiments. A two-tailed Students' t test compares the treated populations. *P ≤0.05. (**c**) BT474 cells treated with either TGFβ (100 pM) or SHP099 (1 or 5 μM) or both for 24 or 48 hours. Lystates were probed with indicated antibodies. (**d**) Quantification of panel C comparing phospho-SMAD2 to corresponding SMAD2. Density was evaluated with IMAGE J. Bars represent mean ± SD from 3 independent experiments. A two-tailed student's t test compares the treated populations. *P ≤0.05. (**e**) LU65 cells treated with either TGFβ (100 pM) or SHP099 (1 or 5 μM) or both for 24 or 48 hours. Lysates were probed with indicated antibodies. (**f**) H358 cells treated with either TGFβ (100 pM) or SHP099 (1 or 5 μM) or both for 24 or 48 hours. Lysates were probed with indicated antibodies. (**g**) TGFβ responsive luciferase (CAGA luciferase) of H358 cells stimulated overnight with TGFβ (100 pM) or MEK162 (1 μM) or GDC-0068 (1 μM) or in combination. Lysates were collected and luciferase measured by a luminometer. Error bars represent SD of triplicates. Experiments are representative of three independent experiments. (**h**) TGFβ responsive luciferase (CAGA luciferase) of H1792 cells stimulated overnight with TGFβ (100 pM) or MEK162 (1 μM) or GDC-0068 (1 μM) or in combination. Lysates were collected and luciferase measured by a luminometer. Error bars represent SD of triplicates. Experiments are representative of three independent experiments. *P ≤ 0.05,**P ≤0.01 as determined by Student's t-test. (**i**) TGFβ responsive luciferase (CAGA luciferase) of 293T cells stimulated overnight with TGFβ (100 pM) or MEK162 (1 μM) or GDC-0068 (1 μM) or in combination. Lysates were collected and luciferase measured by a luminometer. Error bars represent SD of triplicates. Experiments are representative of three independent experiments. ****P ≤0.0001 as determined by Student's t-test. (**j**) TGFβ responsive luciferase (CAGA luciferase) of H358 cells transfected with shSHP2 or a hairpin targeting GFP were stimulated where indicated with BMP7 (50 μg/μl) overnight before lysis. Error bars represent SD of triplicates. Experiments are representative of three independent experiments. **P ≤0.01 as determined by Student's t-test.

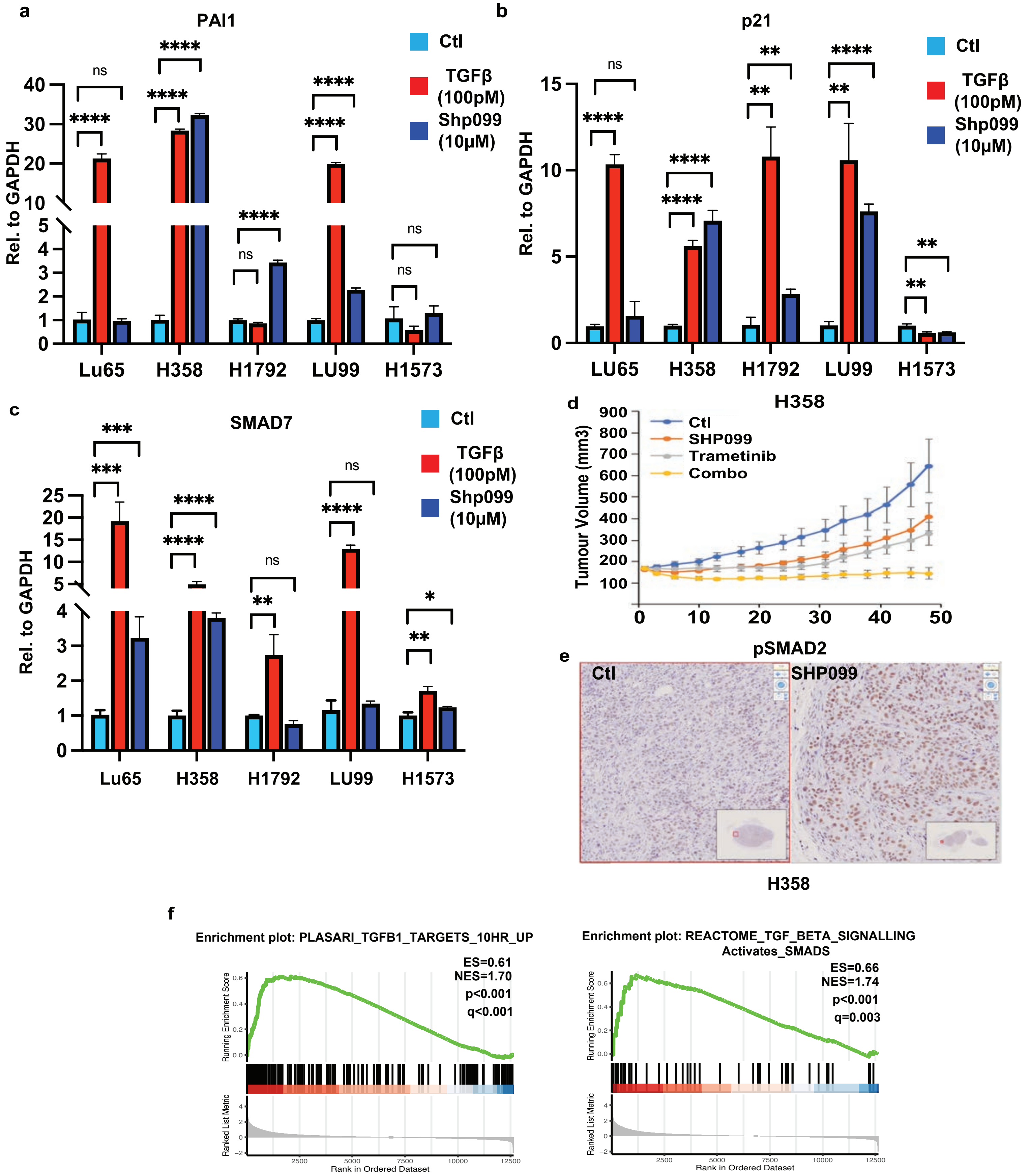

Supplementary Figure 4. SHP2 induces TGFβ pathway activation in KRAS mutant lung cancer.

(**a-c**) LU65, H358, H1792, or LU99 cells were stimulated with TGFβ (100 pM) or SHP099 (10 μM) as indicated for 3 hours. PAI1 (**a**), p21 (**b**), or SMAD7 (**c**) mRNA levels relative to GAPDH are shown as evaluated by quantitative real time PCR. Data are shown as the mean ± SD of triplicate samples from a representative experiment performed two times. *P ≤0.05, **P ≤0.01, ***P ≤0.001, ****P ≤0.0001 as determined by Student's t-test. (**d**) Quantification of nude mice bearing xenograft tumours of H358 cells treated with SHP099 (75 mg/kg), Trametinib (0.3 mg/kg), or the combination for 50 days. Points indicate mean tumour volume, bars, SE. (**e**) Tumours were harvested at day 50 and analyzed by immunohistochemistry for the phosphorylation of SMAD2. Representative images are shown. (**f**) Gene set enrichment analysis of TGFβ (PLASARI_TGFB1_TARGETS_10HR_UP and REACTOME_TGF_ BETA_SIGNALLING_ ACTIVATES_SMADS,) gene set signatures, extrapolated from the GSE109270 data set derived from five vehicle- and seven SHP099-treated patient derived xenograft tumours. Enrichment scores (ESs), normalized enrichment scores (NESs), P values, and FDR are reported.

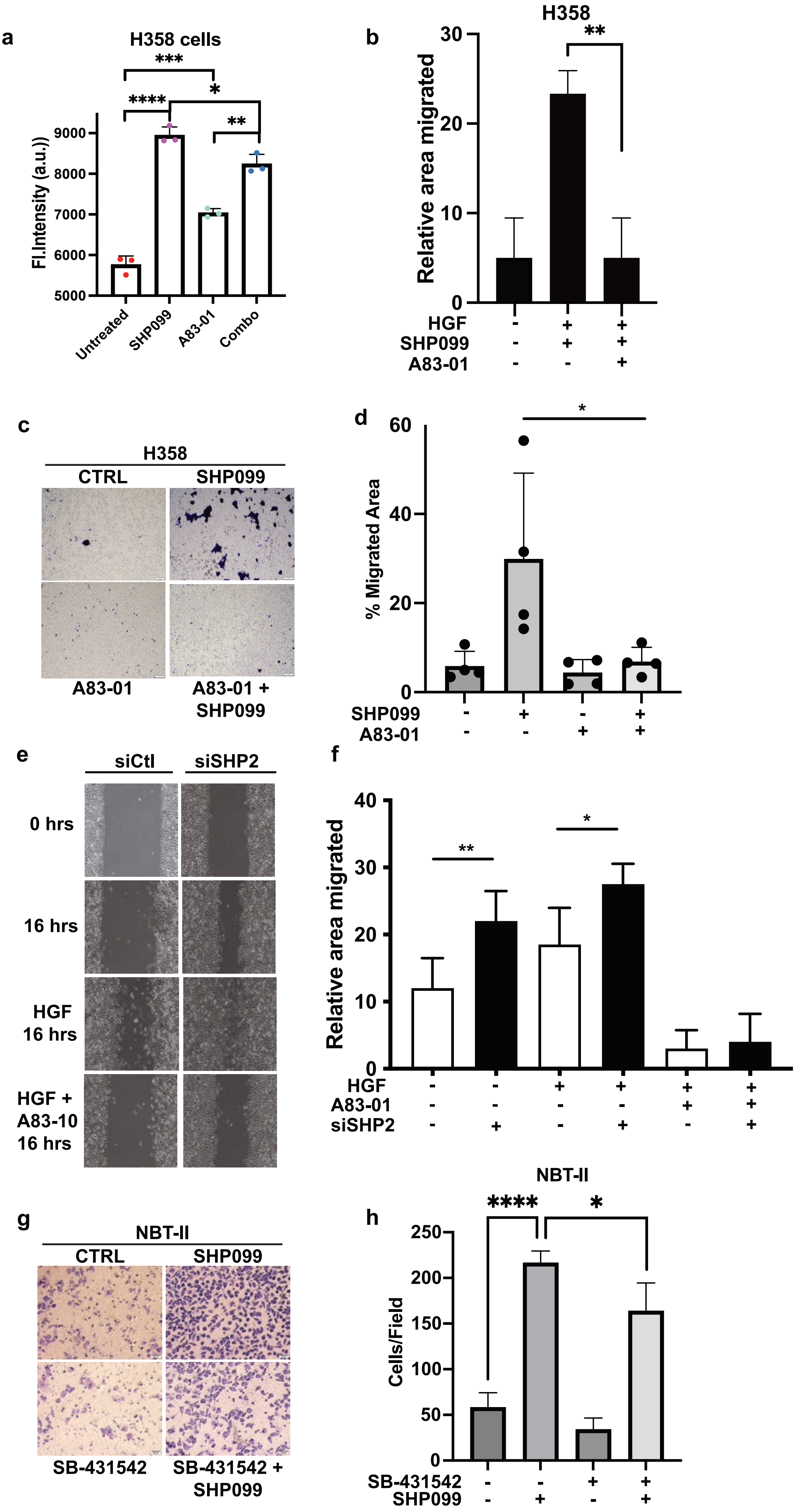
a
H358 cells
***
*
****
**
Fl.Intensity (a.u.))
9000
8000
7000
6000
5000
Untreated
SHP099
A83-01
Combo
b
H358
**
Relative area migrated
30
20
10
0
HGF - + +
SHP099 - + +
A83-01 - - +
c
H358
CTRL
SHP099
A83-01
A83-01 +
SHP099
d
*
% Migrated Area
60
40
20
0
SHP099 - + - +
A83-01 - - + +
e
siCtl
siSHP2
0 hrs
16 hrs
HGF
16 hrs
HGF +
A83-10
16 hrs
f
**
*
Relative area migrated
40
30
20
10
0
HGF - - + + + +
A83-01 - - - - + +
siSHP2 - + - + - +
g
NBT-II
CTRL
SHP099
SB-431542
SB-431542 +
SHP099
h
NBT-II
****
*
Cells/Field
250
200
150
100
50
0
SB-431542 - - + +
SHP099 - + - +

Supplementary Figure 5. TGFβ inhibition circumvents SHP2 inhibitor induced TGFβ responses.

(**a**) Quantification of H358 cells similar to figure 5b,c. Control (red), SHP099 (10 µM) (purple), A83-01 (10 µM) (green) or the combination A83-01 (10 µM) plus SHP099 (10 µM) (blue). Error bars in A represent SD of three independent experiments. *P ≤0.05,**P ≤0.01, ***P≤0.001, **** P ≤0.0001 as determined by Student’s t-test. (**b**) Migration assay in H358 cells similar to 5E. Percentage of migrated area was determined with respect to control (0 h) and a graph was plotted. **P ≤0.01 using Student’s t-test. Data are mean ± SD from three random fields. Data are representative of three independent experiments with similar results. (**c**) Transwell assay of H358 cells treated with SHP099 (10 µM), or in combination with or without A83-01(10 µM) for 16 hours prior to fixation and crystal violet staining (scale bars 100 µm). (**d**) Graph represents percent number of migrated cells taken from four different random fields from panel (**c**). Data are mean SD of triplicate samples from a representative experiment performed three times. *P≤0.05 using Student’s t-test. (**e**) NBT-II cells transduced with siRNA targeting SHP2 or relevant controls were plated for scratch assay and treated with either DMSO, HGF (8 µM), or HGF (8 µM) in combination with A-83-01 (10 µM), panels show migration at 0 and 16 hours. Representative images are shown (scale bars, 200 µm). (**f**). Graph extrapolated from panel E represents percentage of migrated area determined with respect to control (0 h) and a graph was plotted. **P <0.01 using Student’s t-test. Data are mean ± SD from three random fields. Data are representative of three independent experiments with similar results. *P ≤0.05, **P ≤0.01 as determined by Student’s t-test. (**g**) Transwell assay of NBT-II cells treated with SHP099 (5 µM), SB-431542 (10 µM), or in combination for 48 hours prior to fixation and crystal violet staining (scale bars 100 µm). (**h**) Graph represents average number of migrated cells taken from 4 different random fields from panel (**g**). Data are mean ± SD of triplicate samples from a representative experiment performed three times. *P≤0.05, ****P≤0.0001 using Student’s t-test..

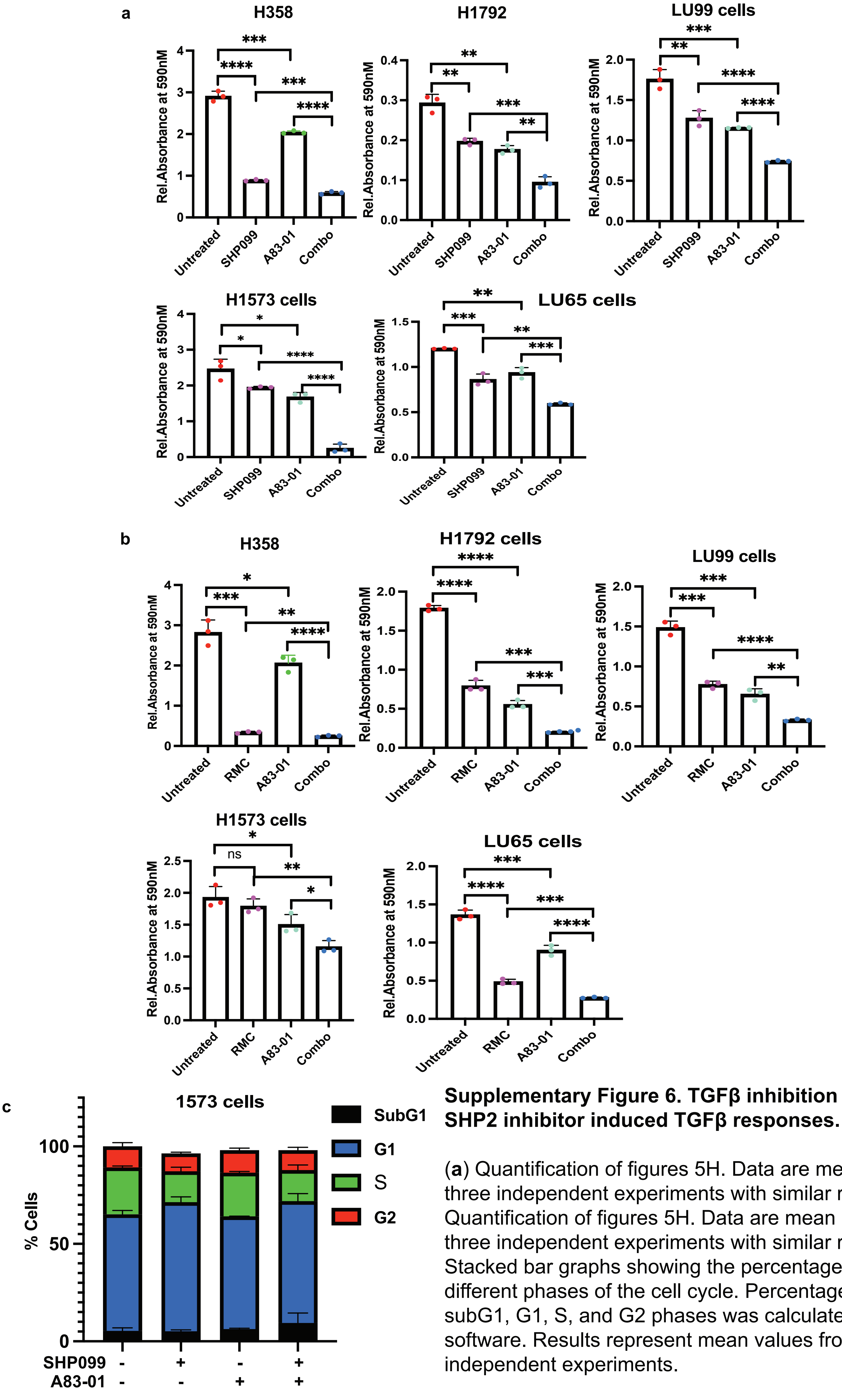

Supplementary Figure 6. TGFβ inhibition circumvents SHP2 inhibitor induced TGFβ responses.

(**a**) Quantification of figures 5H. Data are mean ± SD from three independent experiments with similar results. (**b**) Quantification of figures 5H. Data are mean ± SD from three independent experiments with similar results. (**c**) Stacked bar graphs showing the percentage of cells in different phases of the cell cycle. Percentage of cells in subG1, G1, S, and G2 phases was calculated with Flowjo software. Results represent mean values from three independent experiments.

a

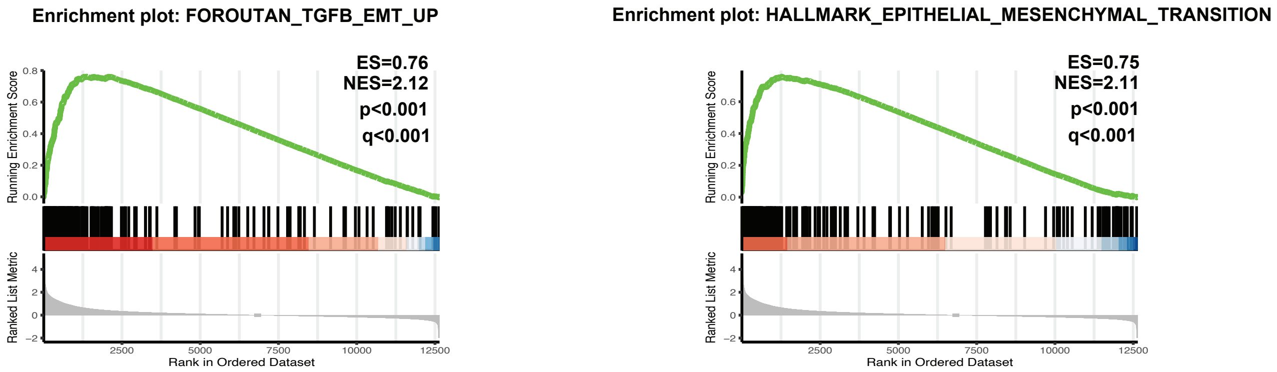

Supplementary Figure 7. SHP099 induces EMT

(**a**) Gene set enrichment analysis of EMT (FOUROUTAN_TGFB_EMT_UP, AND HALL-MARK_ EPITHE- LIAL_MESENCHYMAL_TRANSITION) gene set signatures, extrapolat-ed from the GSE109270 data set derived from five vehicle- and seven SHP099-treated patient-derived xenograft tumours. Enrichment scores (ESs), normalized enrichment scores (NESs), P values, and FDR are reported.

Supplementary Figure 8: Raw data images.

Figure 1D

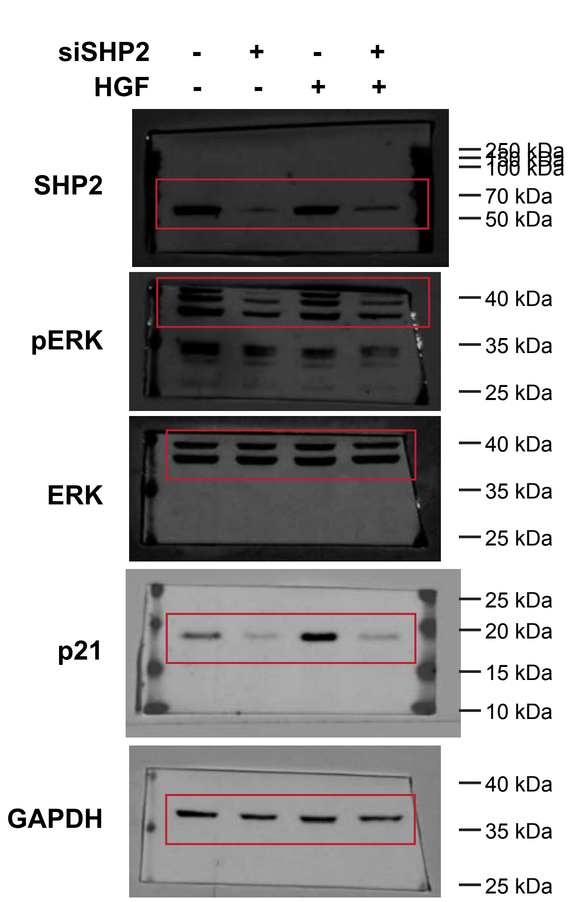

Figure 1I

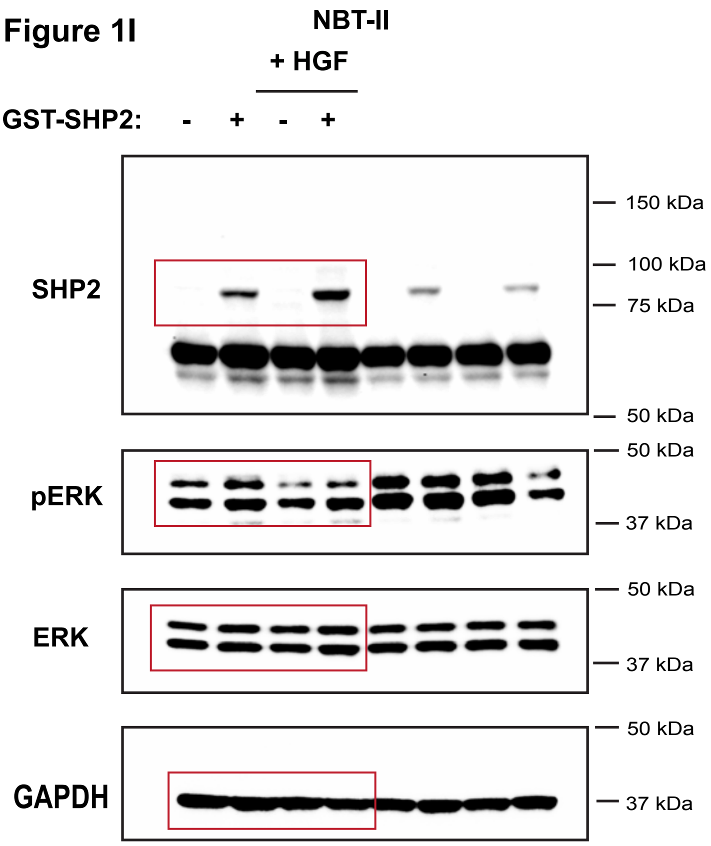

Figure 2C

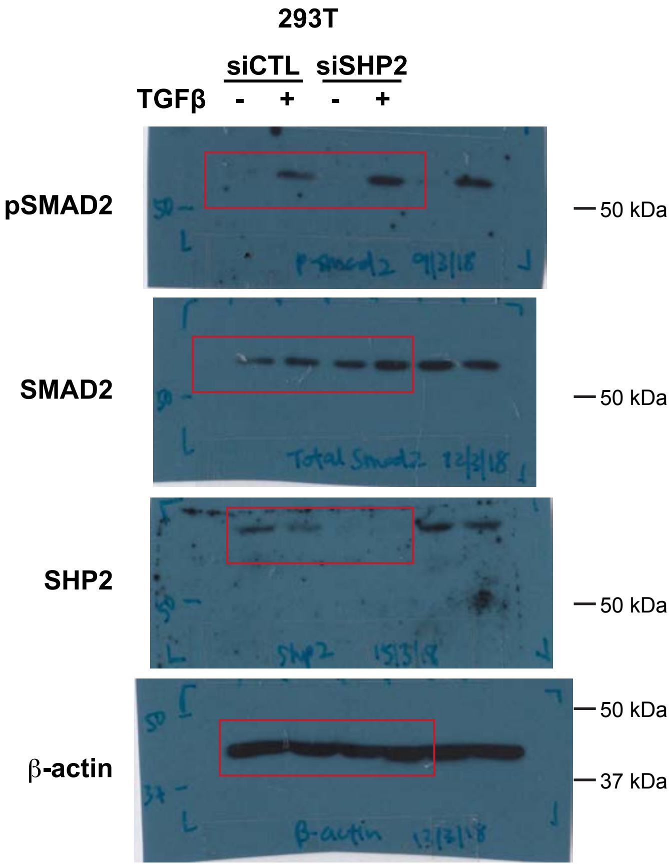

Figure 2E

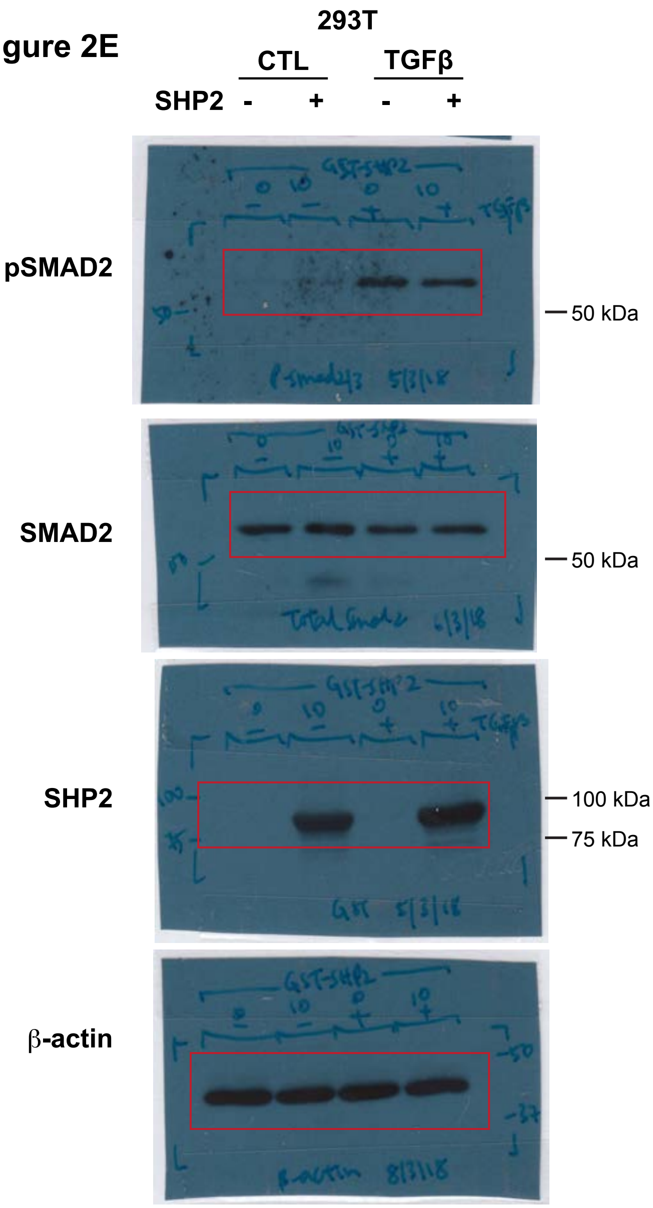

Figure 2G

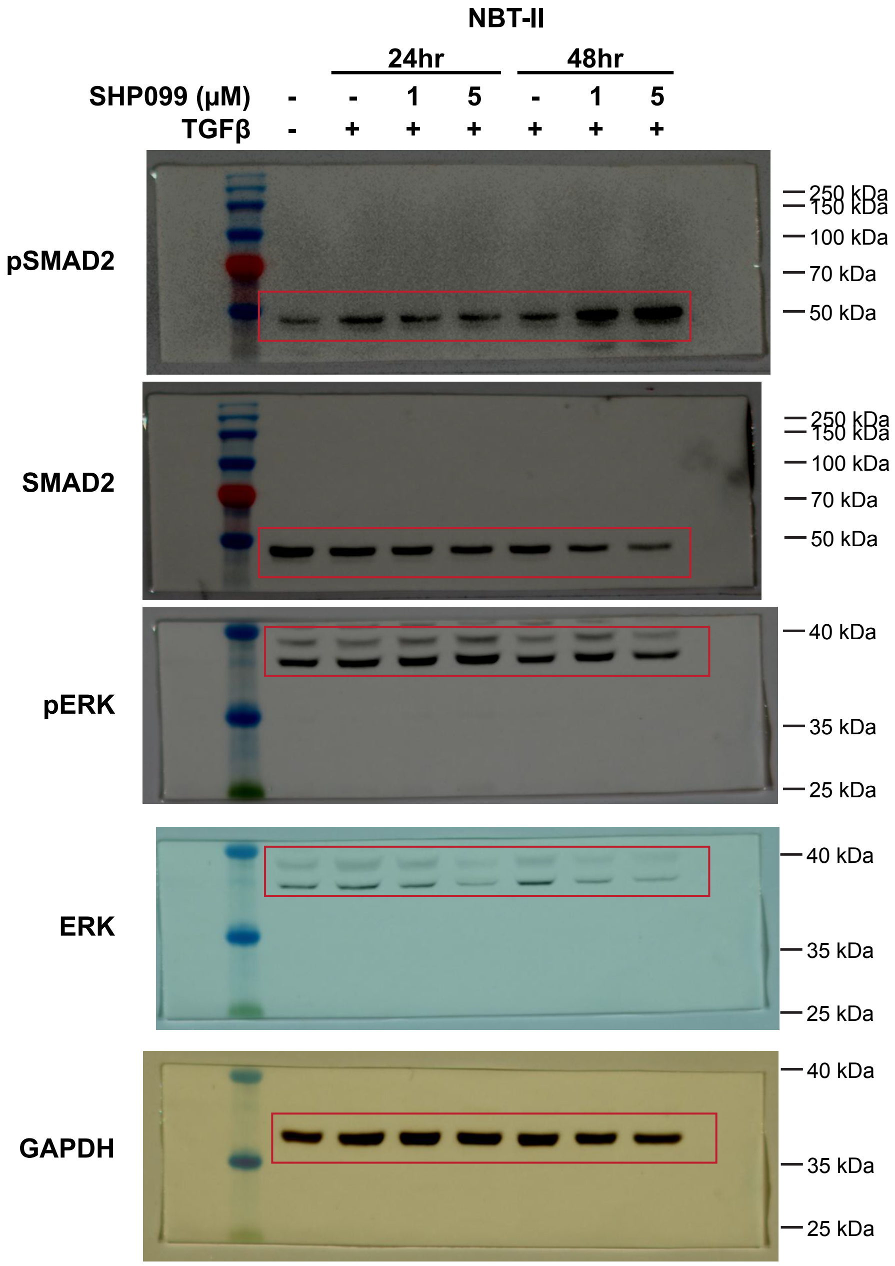
NBT-II
24hr
48hr
SHP099 (μM) - - 1 5 - 1 5
TGFβ - + + + + + +
pSMAD2
SMAD2
pERK
ERK
GAPDH
250 kDa
150 kDa
100 kDa
70 kDa
50 kDa
40 kDa
35 kDa
25 kDa

Figure 4A

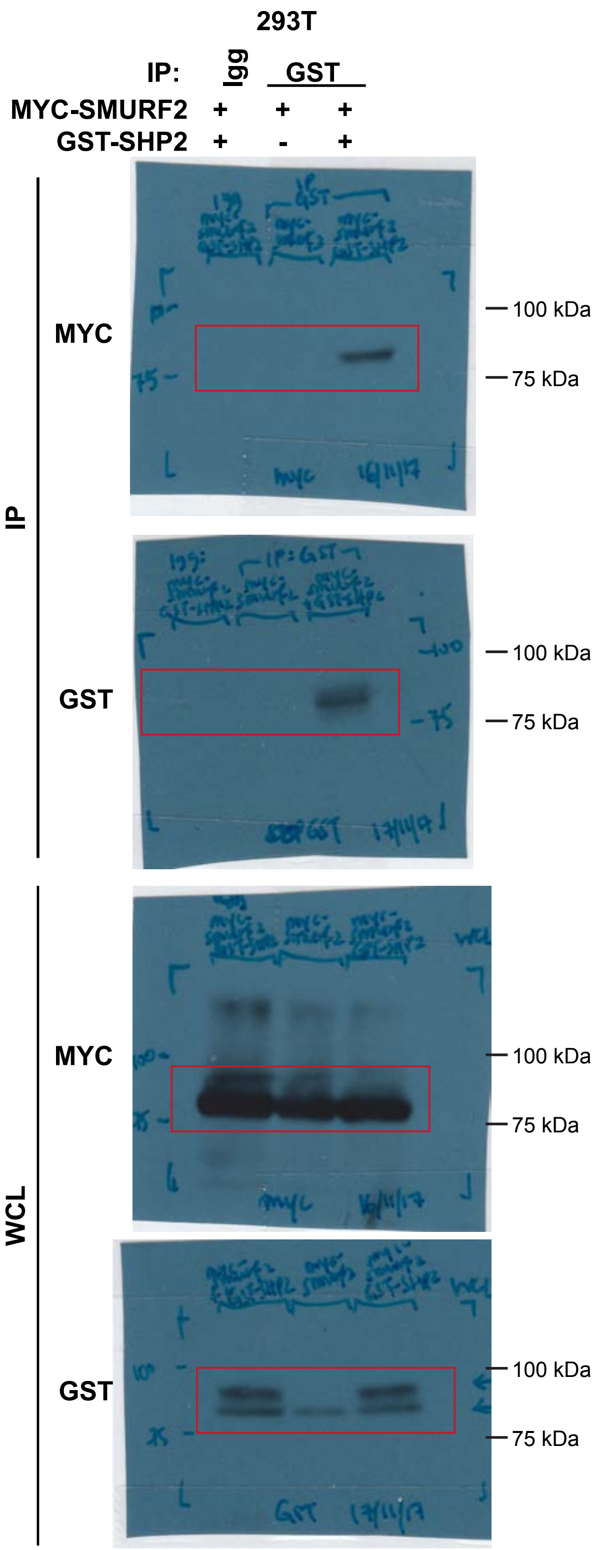

Figure 4B

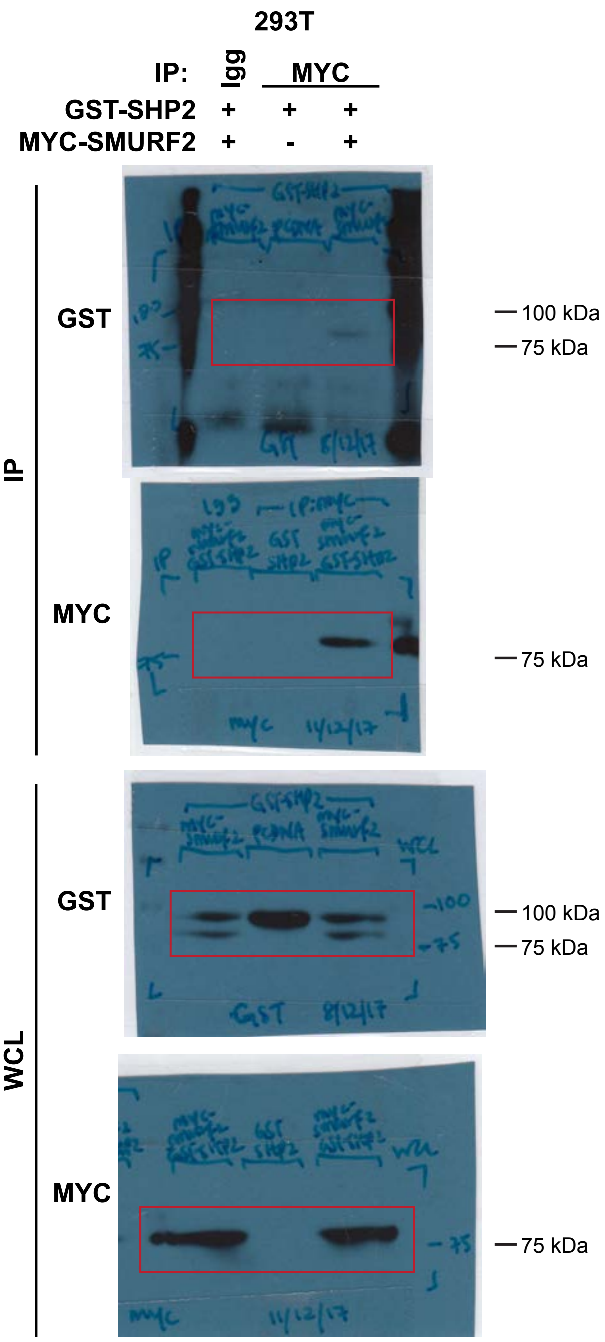

Figure 4C

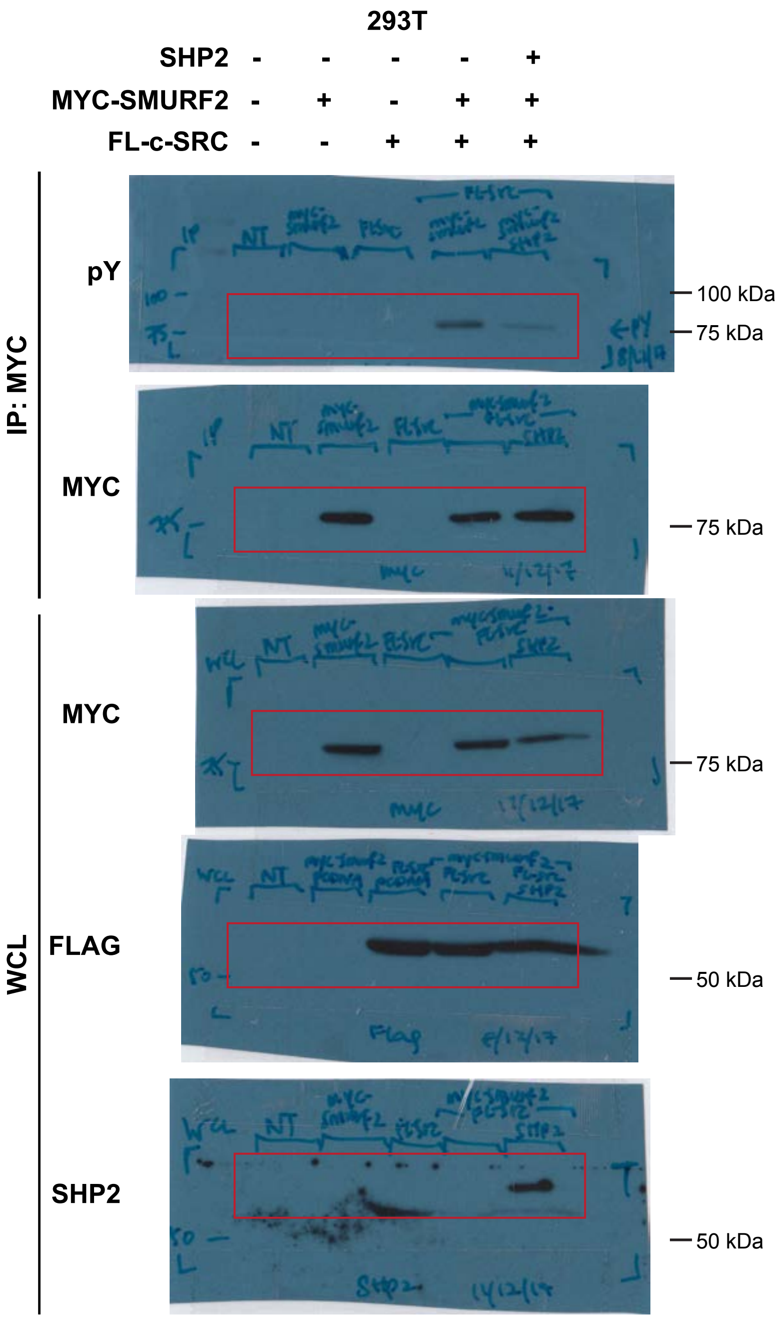

293T
SHP2 - - - - +
MYC-SMURF2 - + - + +
FL-c-SRC - - + + +
IP: MYC
pY
MYC
WCL
MYC
FLAG
SHP2
100 kDa
75 kDa
75 kDa
75 kDa
50 kDa
50 kDa

Figure 4D

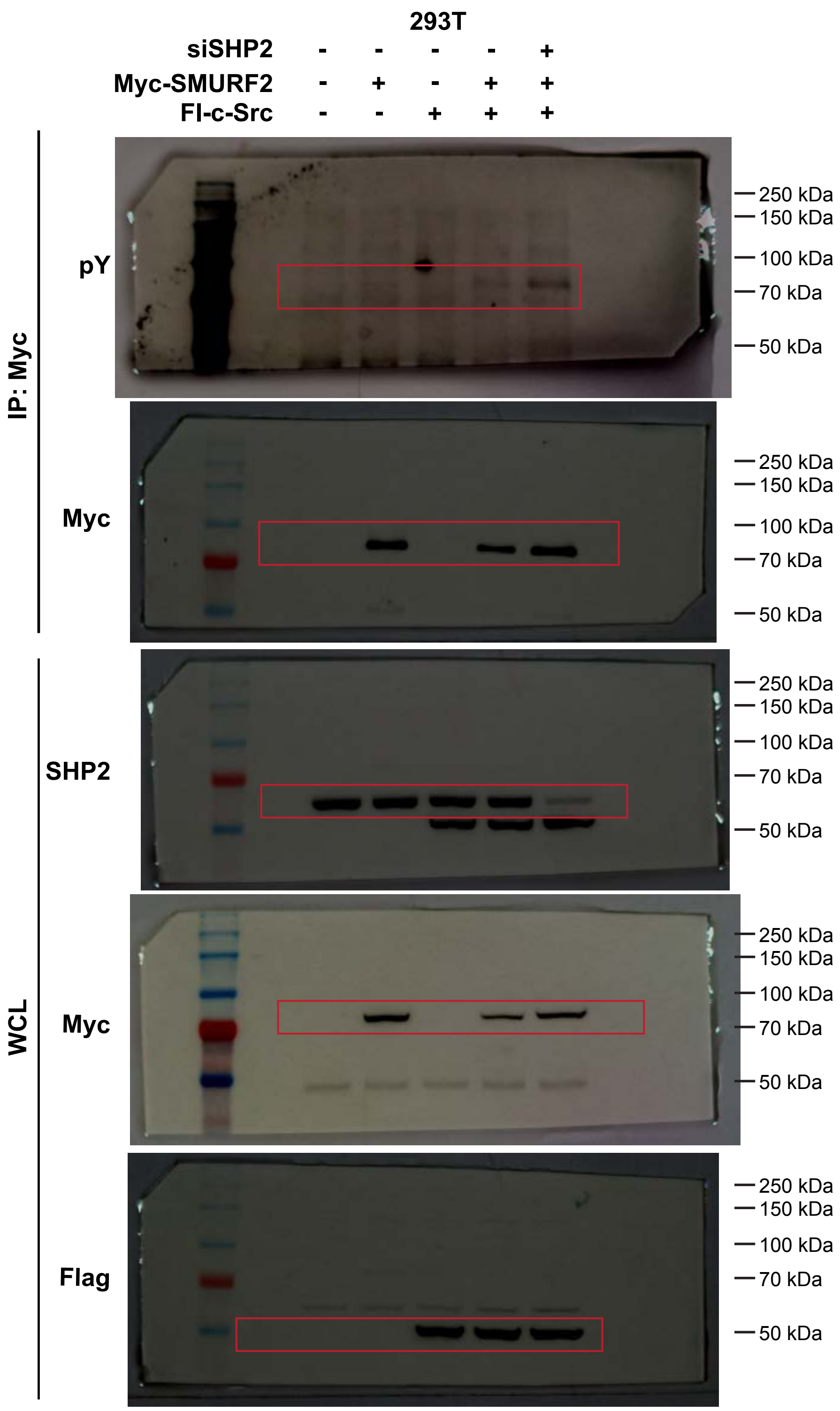
293T
siSHP2 - - - - +
Myc-SMURF2 - + - + +
Fl-c-Src - - + + +
IP: Myc
pY
Myc
WCL
SHP2
Myc
Flag
250 kDa
150 kDa
100 kDa
70 kDa
50 kDa

Figure 4E

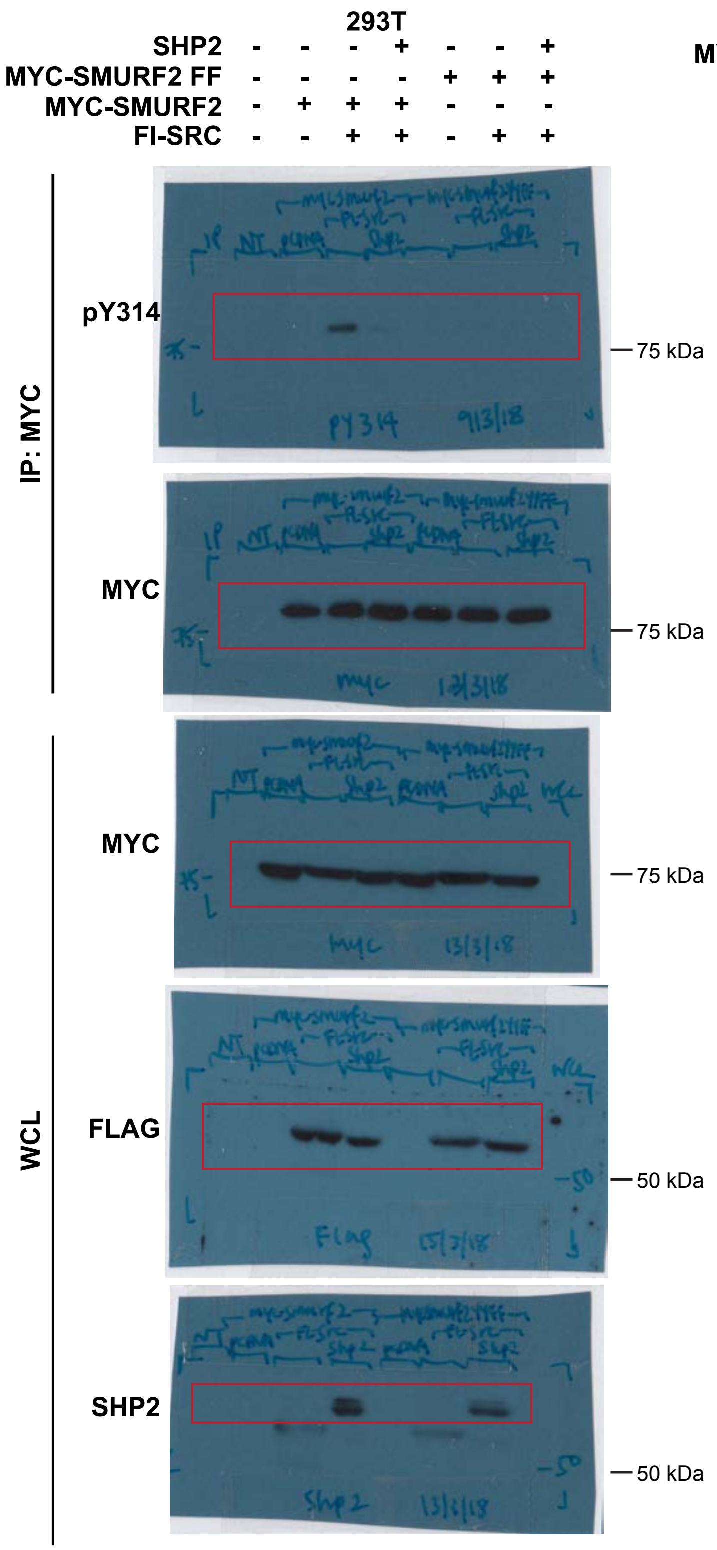

Figure 4F

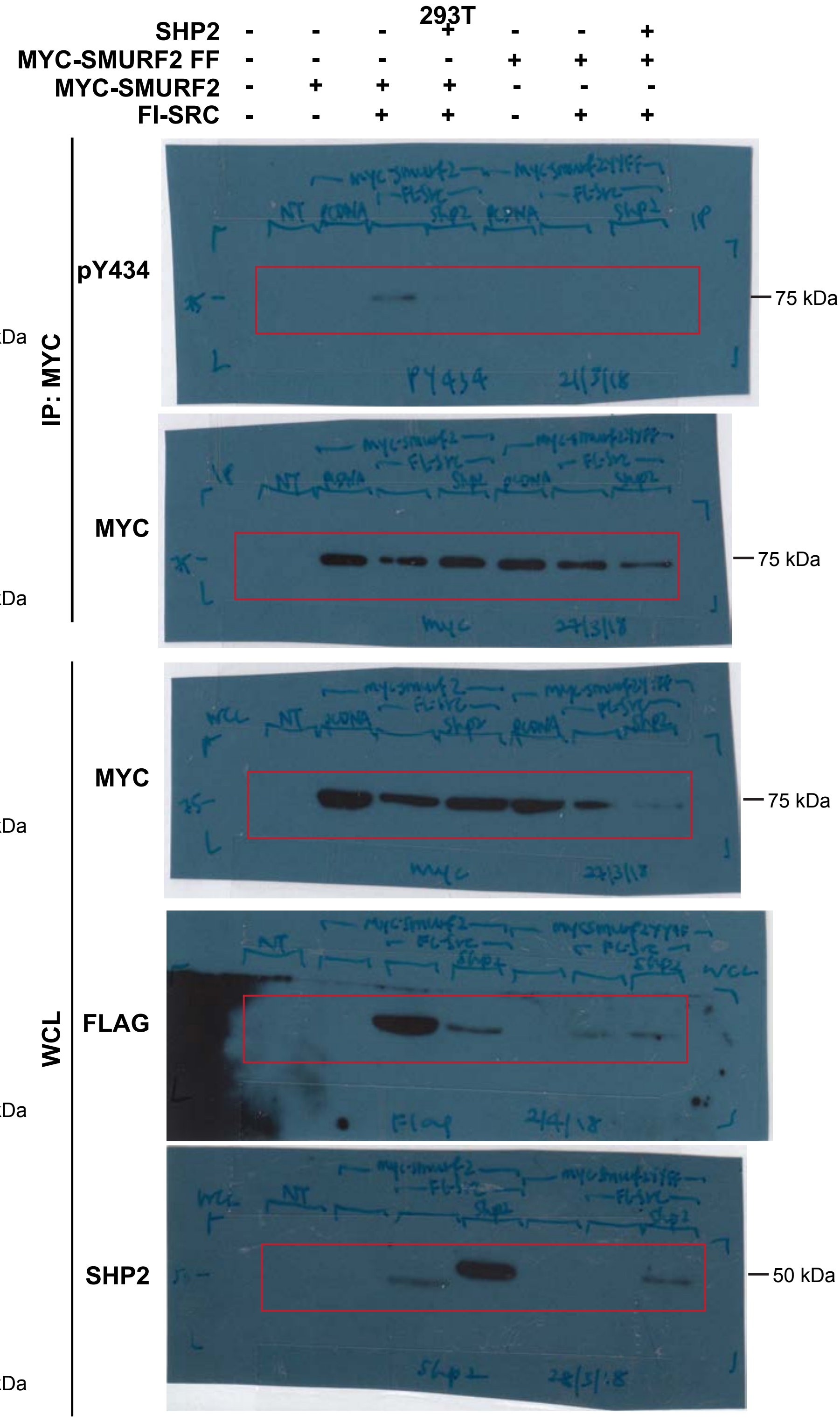

Figure 4G

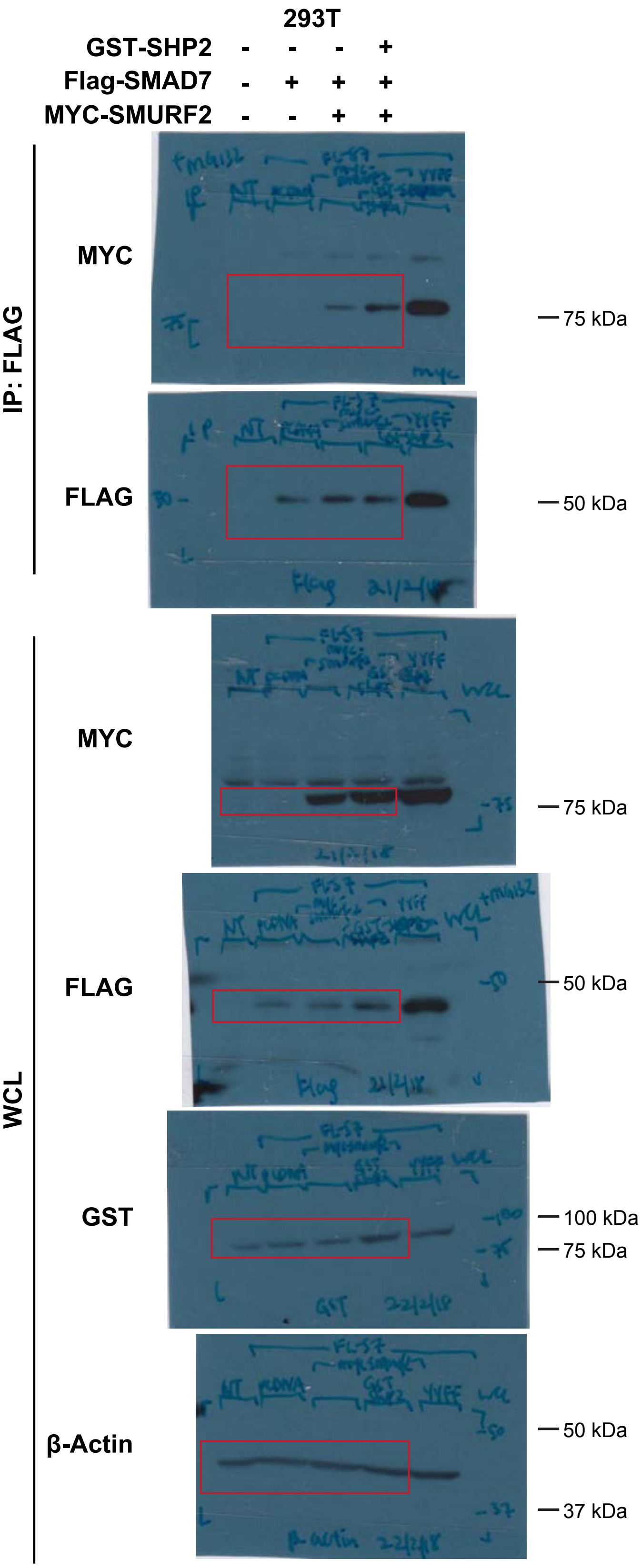

Figure 4H

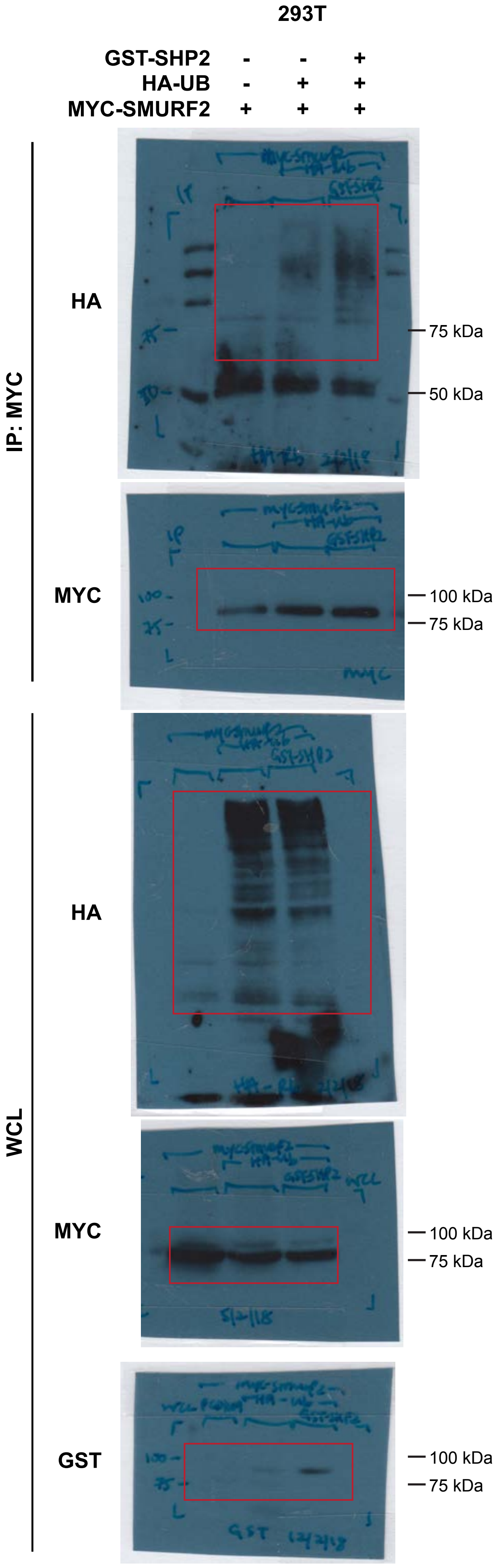

Figure S1C

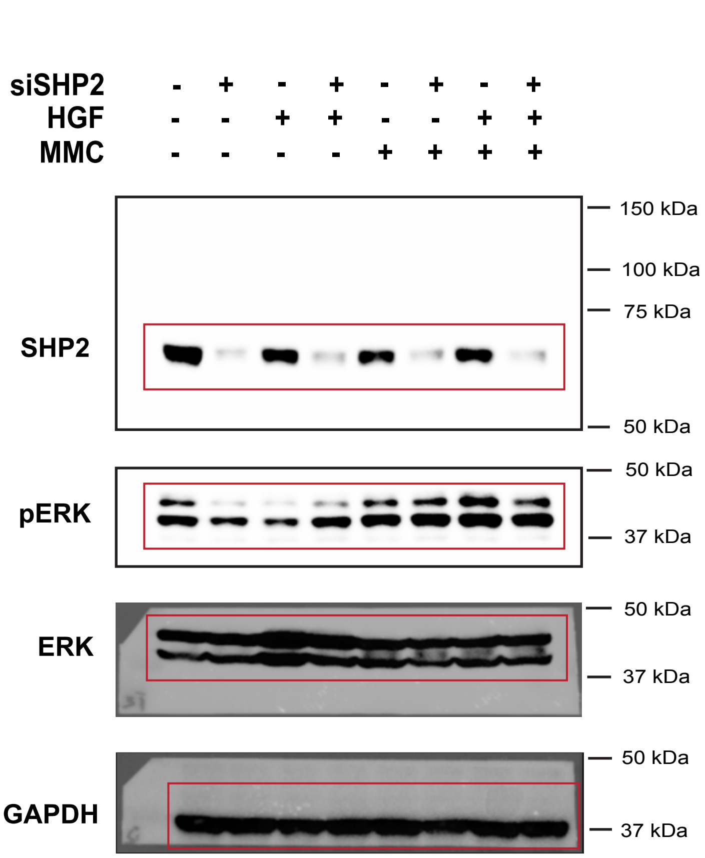

Figure S3A

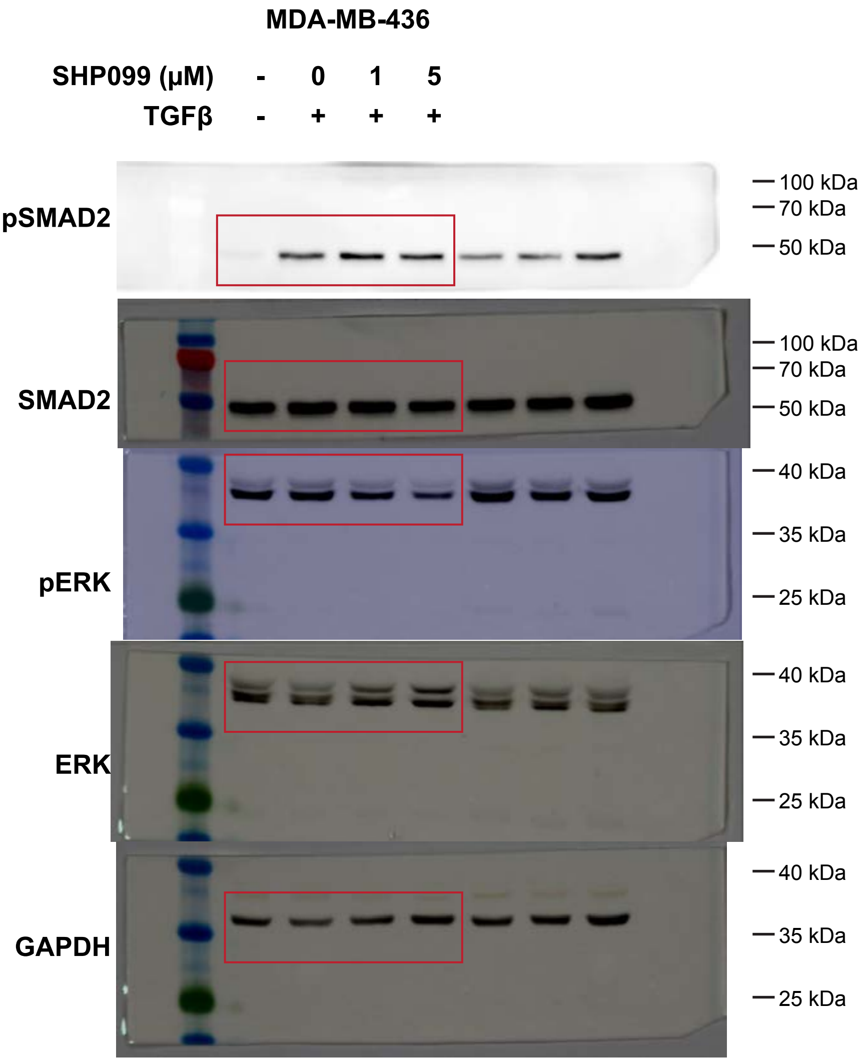

Figure S3C

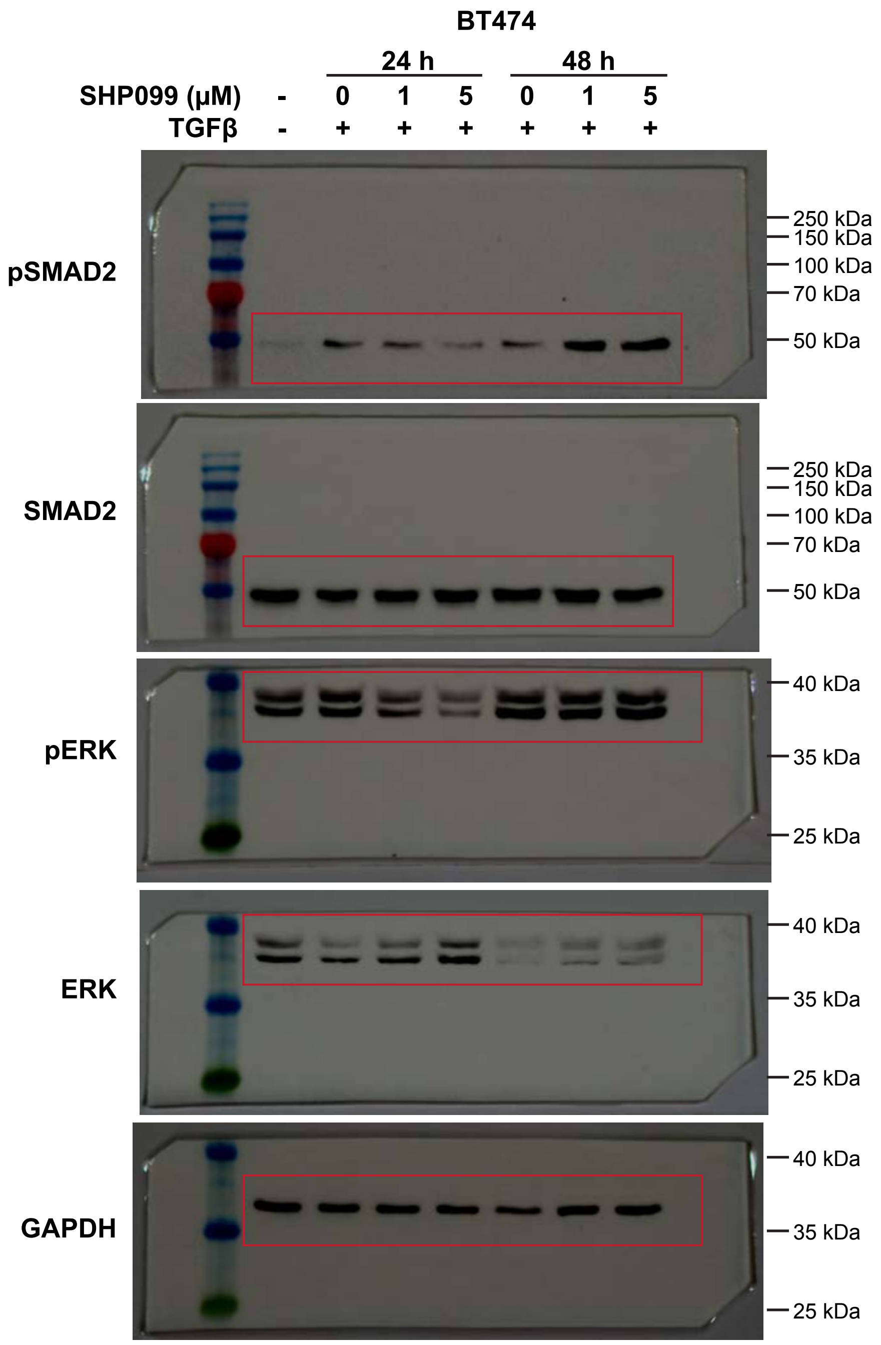
BT474
24 h
48 h
SHP099 (μM) - 0 1 5 0 1 5
TGFβ - + + + + + +
pSMAD2
SMAD2
pERK
ERK
GAPDH
250 kDa
150 kDa
100 kDa
70 kDa
50 kDa
40 kDa
35 kDa
25 kDa

Figure S3E/F

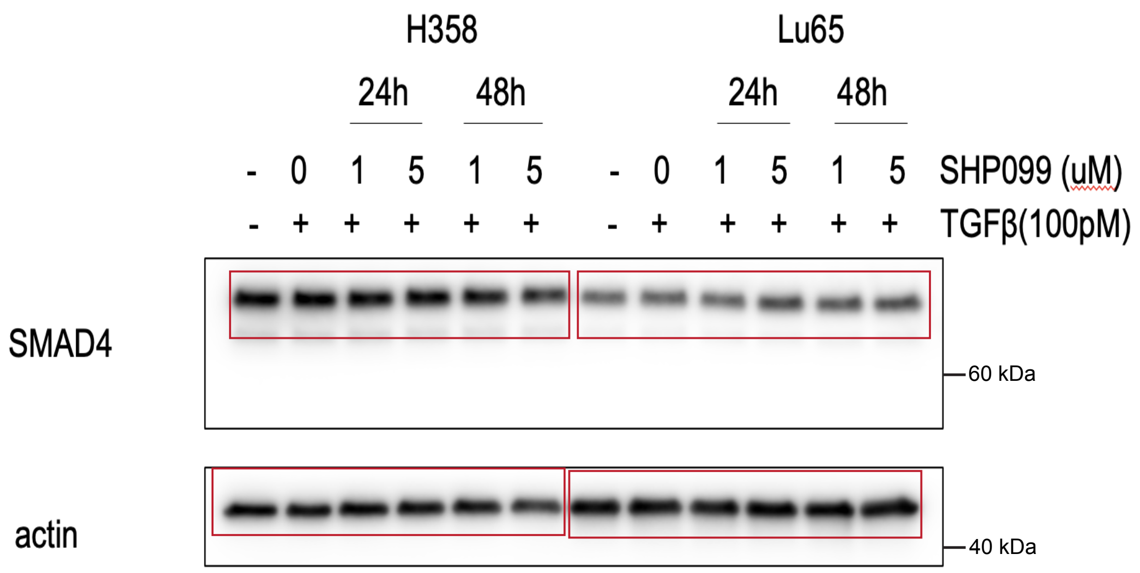
H358
Lu65
24h
48h
24h
48h
- 0 1 5 1 5 - 0 1 5 1 5 SHP099 (uM)
- + + + + + - + + + + + TGFβ(100pM)
SMAD4
60 kDa
actin
40 kDa

Supplementary Table 1

Gene	Description	% WHR
Atp7a	ATPase, Cu++ transporting, alpha polypeptide	169
Dusp3	dual specificity phosphatase 3	169
Ppp2r2b	protein phosphatase 2 (formerly 2A), regulatory subunit B (PR 52), beta isoform	163
Atp7b	ATPase, Cu++ transporting, beta polypeptide	156
Ppp2r2c	protein phosphatase 2 (formerly 2A), regulatory subunit B, gamma isoform	150
Ptpn13	protein tyrosine phosphatase, non-receptor type 13	149
LOC499736	similar to Dual specificity protein phosphatase 14 (Mitogen-activated protein kinase phosphatase 6)	148
Dut	deoxyuridine triphosphatase	147
Ppp1r3c	protein phosphatase 1, regulatory (inhibitor) subunit 3C	146
Ptpn11	protein tyrosine phosphatase, non-receptor type 11	146
Ppp2r5c	protein phosphatase 2, regulatory subunit B' gamma isoform	142
Styxl1	serine/threonine/tyrosine interacting-like 1	139
Ptprn2	protein tyrosine phosphatase, receptor type, N polypeptide 2	138
Pfkfb2	6-phosphofructo-2-kinase/fructose-2,6-biphosphatase 2	137
Ppp1r9a	protein phosphatase 1, regulatory (inhibitor) subunit 9A	135
Psph	phosphoserine phosphatase	135
Ppp1r12b	protein phosphatase 1, regulatory (inhibitor) subunit 12B	134
Dolpp1	dolichyl pyrophosphate phosphatase 1	134
Sbf1	SET binding factor 1	133
Ptpn23	protein tyrosine phosphatase, non-receptor type 23	133
Pten	phosphatase and tensin homolog	132
Ptprb	protein tyrosine phosphatase, receptor type, B	132
Fbp2	fructose-1,6-bisphosphatase 2	131
Ppp4r1	protein phosphatase 4, regulatory subunit 1	131
Pdp2	pyruvate dehydrogenase phosphatase isoenzyme 2	131
Ppm1a	protein phosphatase 1A, magnesium dependent, alpha isoform	131
Ppp1ca	protein phosphatase 1, catalytic subunit, alpha isoform	131
Lhpp	phospholysine phosphohistidine inorganic pyrophosphate phosphatase	131
Ppp1r14d	protein phosphatase 1, regulatory (inhibitor) subunit 14D	131
Dusp13	dual specificity phosphatase 13	130
Ptprf	protein tyrosine phosphatase, receptor type, F	130
Ppef1	protein phosphatase, EF-hand calcium binding domain 1	130
Ptpro	protein tyrosine phosphatase, receptor type, O	129
Inpp4b	inositol polyphosphate-4-phosphatase, type II	129
Ptpn14	protein tyrosine phosphatase, non-receptor type 14	128
Ppp2cb	protein phosphatase 2 (formerly 2A), catalytic subunit, beta isoform	128
Phospho1	phosphatase, orphan 1	128
Ppm1g	protein phosphatase 1G (formerly 2C), magnesium-dependent, gamma isoform	127
Dusp6	dual specificity phosphatase 6	127
Ppp2r4	protein phosphatase 2A activator, regulatory subunit 4	127
Enpp1	ectonucleotide pyrophosphatase/phosphodiesterase 1	126
Ptpn21	protein tyrosine phosphatase, non-receptor type 21	126
G6pc	glucose-6-phosphatase, catalytic subunit	125
Dusp19	dual specificity phosphatase 19	125
Ppap2a	phosphatidic acid phosphatase type 2A	140
LOC499736	similar to Dual specificity protein phosphatase 14 (Mitogen-activated protein kinase phosphatase 6)	129
Impa2	inositol (myo)-1(or 4)-monophosphatase 2	127

Mtmr2	myotubularin related protein 2	127
Nt5e	5' nucleotidase, ecto	126
Fbp1	fructose-1,6- biphosphatase 1	124
Ptpra	protein tyrosine phosphatase, receptor type, A	124
Ppp2r3a	protein phosphatase 2 (formerly 2A), regulatory subunit B'', alpha	124
Ppp1cb	protein phosphatase 1, catalytic subunit, beta isoform	124
Ppef2	protein phosphatase, EF hand calcium-binding domain 2	124
Ppp3ca	protein phosphatase 3 (formerly 2B), catalytic subunit, alpha isoform	124
Dusp10	dual specificity phosphatase 10	123
Ptprm	protein tyrosine phosphatase, receptor type, M	123
Ppm1b	protein phosphatase 1B, magnesium dependent, beta isoform	123
Ptprs	protein tyrosine phosphatase, receptor type, S	122
Ppm1l	protein phosphatase 1 (formerly 2C)-like	122
Dusp23	dual specificity phosphatase 23	122
Alpl	alkaline phosphatase, liver/bone/kidney	122
Ppp3cc	protein phosphatase 3, catalytic subunit, gamma isoform	121
Ppp1r3a	protein phosphatase 1, regulatory (inhibitor) subunit 3A	121
Ptp4a3	protein tyrosine phosphatase 4a3	121
Ppp2r2d	protein phosphatase 2, regulatory subunit B, delta isoform	121
Dusp11	dual specificity phosphatase 11 (RNA/RNP complex 1-interacting)	120
Ptplb	protein tyrosine phosphatase-like (proline instead of catalytic arginine),	120
Ppm1d	protein phosphatase 1D magnesium-dependent, delta isoform	120
Itpa	inosine triphosphatase (nucleoside triphosphate pyrophosphatase)	120
Ilkap	integrin-linked kinase-associated serine/threonine phosphatase 2C	120
Ppm2c	protein phosphatase 2C, magnesium dependent, catalytic subunit	120
Acp1	acid phosphatase 1, soluble	120
Ptprh	protein tyrosine phosphatase, receptor type, H	119
Ppp2r1b	protein phosphatase 2 (formerly 2A), regulatory subunit A (PR 65), beta isoform	119
Mprip	myosin phosphatase Rho interacting protein	119
Ppm1f	protein phosphatase 1F (PP2C domain containing)	119
Ptpn6	protein tyrosine phosphatase, non-receptor type 6	118
Inpp5a	inositol polyphosphate-5-phosphatase A	118
Ppm1e	protein phosphatase 1E (PP2C domain containing)	118
Ppp2ca	protein phosphatase 2 (formerly 2A), catalytic subunit, alpha isoform	118
Tpte	transmembrane phosphatase with tensin homology	118
Ptprc	protein tyrosine phosphatase, receptor type, C	118
Ppp1r10	protein phosphatase 1, regulatory subunit 10	118
Sirpa	signal-regulatory protein alpha	117
Ptpn9	protein tyrosine phosphatase, non-receptor type 9	117
Dusp18	dual specificity phosphatase 18	117
Pfkfb3	6-phosphofructo-2-kinase/fructose-2,6-biphosphatase 3	117
Rngtt	RNA guanylyltransferase and 5'-phosphatase	116
Enpp2	ectonucleotide pyrophosphatase/phosphodiesterase 2	116
Dusp7	dual specificity phosphatase 7	116
Ppp2r1a	protein phosphatase 2 (formerly 2A), regulatory subunit A, alpha isoform	116
Ppp5c	protein phosphatase 5, catalytic subunit	115
Ppp2r5a	protein phosphatase 2, regulatory subunit B', alpha isoform	115
Ptprd	protein tyrosine phosphatase, receptor type, D	115

Dusp4	dual specificity phosphatase 4	114
Ppp6c	protein phosphatase 6, catalytic subunit	113
Ppme1	protein phosphatase methylesterase 1	113
Ptpn7	protein tyrosine phosphatase, non-receptor type 7	113
Ctdp1	CTD (carboxy-terminal domain, RNA polymerase II, polypeptide A) phosphatase, subunit 1	113
Ptpn2	protein tyrosine phosphatase, non-receptor type 2	112
Ptprg	protein tyrosine phosphatase, receptor type, G	111
Ppp2r2a	protein phosphatase 2 (formerly 2A), regulatory subunit B, alpha isoform	111
Mtmr4	myotubularin related protein 4	110
Ptpn4	protein tyrosine phosphatase, non-receptor type 4	110
Mtmr6	myotubularin related protein 6	110
Ptpn1	protein tyrosine phosphatase, non-receptor type 1	110
Ptpn22	protein tyrosine phosphatase, non-receptor type 22 (lymphoid)	109
Mtmr3	myotubularin related protein 3	109
Ppp1r15b	protein phosphatase 1, regulatory (inhibitor) subunit 15b	109
Ppp1r9b	protein phosphatase 1, regulatory subunit 9B	108
Dusp14	dual specificity phosphatase 14	108
Ppp1r14a	protein phosphatase 1, regulatory (inhibitor) subunit 14A	108
Inpp5d	inositol polyphosphate-5-phosphatase D	108
Pfkfb4	6-phosphofructo-2-kinase/fructose-2,6-biphosphatase 4	108
Ppp2r5e	protein phosphatase 2, regulatory subunit B', epsilon isoform	107
Ptpn5	protein tyrosine phosphatase, non-receptor type 5	107
Cdc25b	cell division cycle 25 homolog B (S. pombe)	107
Ppp1cc	protein phosphatase 1, catalytic subunit, gamma isoform	107
Atp2a3	ATPase, Ca++ transporting, ubiquitous	106
Ppp1r14c	protein phosphatase 1, regulatory (inhibitor) subunit 14c	106
Ppap2c	phosphatidic acid phosphatase type 2c	105
Ptp4a2	protein tyrosine phosphatase 4a2	105
Ptpn12	protein tyrosine phosphatase, non-receptor type 12	105
Dusp12	dual specificity phosphatase 12	103
Atp12a	ATPase, H+/K+ transporting, nongastric, alpha polypeptide	99
Ptprr	protein tyrosine phosphatase, receptor type, R	99
Ppp1r1b	protein phosphatase 1, regulatory (inhibitor) subunit 1B	99
Ppp3r1	protein phosphatase 3, regulatory subunit B, alpha isoform (calcineurin B, type I)	99
Ppp1r12c	protein phosphatase 1, regulatory (inhibitor) subunit 12C	99
Ppfia3	protein tyrosine phosphatase, receptor type, f polypeptide (PTPRF), interacting protein (liprin), alpha	98
Ppap2b	phosphatidic acid phosphatase type 2B	97
Ppp1r1a	protein phosphatase 1, regulatory (inhibitor) subunit 1A	97
Inpp1	inositol polyphosphate-1-phosphatase	97
Acp6	acid phosphatase 6, lysophosphatidic	96
Inppl1	inositol polyphosphate phosphatase-like 1	95
Minpp1	multiple inositol polyphosphate histidine phosphatase 1	93
Bpnt1	3'(2'), 5'-bisphosphate nucleotidase 1	92
Tenc1	tensin like C1 domain containing phosphatase (tensin 2)	91
Ppp4c	protein phosphatase 4, catalytic subunit	88
Ptprn	protein tyrosine phosphatase, receptor type, N	88
Inpp5f	inositol polyphosphate-5-phosphatase F	87
Pgam2	phosphoglycerate mutase 2 (muscle)	87

Ppp1r2	protein phosphatase 1, regulatory (inhibitor) subunit 2	87
Dusp8	dual specificity phosphatase 8	86
Dusp2	dual specificity phosphatase 2	86
Ppfia1	protein tyrosine phosphatase, receptor type, f polypeptide (PTPRF), interacting protein (liprin), alpha	85
Ppp1r1c	protein phosphatase 1, regulatory (inhibitor) subunit 1C	85
Ppp1r7	protein phosphatase 1, regulatory (inhibitor) subunit 7	84
Dusp16	dual specificity phosphatase 16	83
Atp2a2	ATPase, Ca++ transporting, cardiac muscle, slow twitch 2	81
Ptpre	protein tyrosine phosphatase, receptor type, E	81
Ptpdc1	protein tyrosine phosphatase domain containing 1	81
Dusp1	dual specificity phosphatase 1	80
Akp3	alkaline phosphatase 3, intestine, not Mn requiring	79
Impa1	Inositol (myo)-1(or 4)-monophosphatase 1	78
Alpi	alkaline phosphatase, intestinal	78
Ppp1r13b	protein phosphatase 1, regulatory (inhibitor) subunit 13B	77
Ppp1r12a	protein phosphatase 1, regulatory (inhibitor) subunit 12A	76
Ppfia2	protein tyrosine phosphatase, receptor type, f polypeptide (PTPRF), interacting protein (liprin), alpha	76
Phpt1	phosphohistidine phosphatase 1	76
Ppp3cb	protein phosphatase 3, catalytic subunit, beta isoform	75
Cdc25a	cell division cycle 25 homolog A (S. pombe)	75
Pdpr	pyruvate dehydrogenase phosphatase regulatory subunit	75
Ptprz1	protein tyrosine phosphatase, receptor-type, Z polypeptide 1	74
Dusp15	dual specificity phosphatase-like 15	74
Inpp4a	inositol polyphosphate-4-phosphatase, type 1	71
Ptp4a1	protein tyrosine phosphatase 4a1	68
Pnkp	polynucleotide kinase 3'-phosphatase	67
Pstpip1	proline-serine-threonine phosphatase-interacting protein 1	67
Inpp5b	inositol polyphosphate-5-phosphatase B	65
G6pc3	glucose 6 phosphatase, catalytic, 3	64
Inpp5j	inositol polyphosphate-5-phosphatase J	63
Dusp22	dual specificity phosphatase 22	62
Sgpp1	sphingosine-1-phosphate phosphatase 1	61
Ppp1r15a	protein phosphatase 1, regulatory (inhibitor) subunit 15A	61
Ppp2r5b	protein phosphatase 2, regulatory subunit B', beta isoform	61
Lppr2	lipid phosphate phosphatase-related protein type 2	58
Pld1	phospholipase D1	57
Atp2a1	ATPase, Ca++ transporting, cardiac muscle, fast twitch 1	57
Mtmr9	myotubularin related protein 9	56
Minpp1	multiple inositol polyphosphate histidine phosphatase 1	55
Ppp1r3b	protein phosphatase 1, regulatory (inhibitor) subunit 3B	55
Cdkn3	cyclin-dependent kinase inhibitor 3	54
Atp2c1	ATPase, Ca++ transporting, type 2C, member 1	54
Ppp1r14b	protein phosphatase 1, regulatory (inhibitor) subunit 14B	51
Cdc25c	cell division cycle 25 homolog C (S. pombe)	47
Acpt	acid phosphatase, testicular	46
Pstpip2	proline-serine-threonine phosphatase-interacting protein 2	43
Synj2	synaptojanin 2	40
Acpp	acid phosphatase, prostate	39

Ctdspl	CTD (carboxy-terminal domain, RNA polymerase II, polypeptide A) small phosphatase-like	39
Ppp1r11	protein phosphatase 1, regulatory (inhibitor) subunit 11	36
Entpd3	ectonucleoside triphosphate diphosphohydrolase 3	31
Pptc7	PTC7 protein phosphatase homolog (S. cerevisiae)	24
Acp2	acid phosphatase 2, lysosomal	20
Enpp3	ectonucleotide pyrophosphatase/phosphodiesterase 3	15
Ppp1r8	protein phosphatase 1, regulatory (inhibitor) subunit 8	0

Supplementary Table 1. siRNA phosphatase screen.

Table represents list of siRNAs transduced into NBT-II cells and treated with HGF. siRNA are ranked according to percent wound healing repair (%WHR).
